# Characterization of gastric cancer-stimulated signaling pathways and function of CTGF in cancer-associated fibroblasts

**DOI:** 10.1186/s12964-023-01396-7

**Published:** 2024-01-02

**Authors:** Kyoung-Min Choi, Boram Kim, Su-Min Lee, Jisoo Han, Ha-Song Bae, Su-Bhin Han, Dagyeong Lee, In-Hye Ham, Hoon Hur, Eunjung Kim, Jae-Young Kim

**Affiliations:** 1 Graduate School of Analytical Science and Technology (GRAST), Chungnam National University, Daejeon, South Korea; 2 Department of Surgery, Ajou University School of Medicine, Suwon, South Korea; 3 Inflamm-Aging Translational Research Center, Ajou University School of Medicine, Suwon, South Korea; 4 AI-Super Convergence KIURI Translational Research Center, Suwon, South Korea; 5 Natural Product Informatics Center, Korea Institute of Science and Technology (KIST), Gangneung, South Korea

**Keywords:** Cancer-associated fibroblasts (CAFs), Tumor microenvironment (TME), Gastric cancer (GC), Connective tissue growth factor (CTGF)

## Abstract

**Background:**

Cancer-associated fibroblasts (CAFs) are key components of the tumor microenvironment (TME) that play an important role in cancer progression. Although the mechanism by which CAFs promote tumorigenesis has been well investigated, the underlying mechanism of CAFs activation by neighboring cancer cells remains elusive. In this study, we aim to investigate the signaling pathways involved in CAFs activation by gastric cancer cells (GC) and to provide insights into the therapeutic targeting of CAFs for overcoming GC.

**Methods:**

Alteration of receptor tyrosine kinase (RTK) activity in CAFs was analyzed using phospho-RTK array. The expression of CAFs effector genes was determined by RT-qPCR or ELISA. The migration and invasion of GC cells co-cultured with CAFs were examined by transwell migration/invasion assay.

**Results:**

We found that conditioned media (CM) from GC cells could activate multiple receptor tyrosine kinase signaling pathways, including ERK, AKT, and STAT3. Phospho-RTK array analysis showed that CM from GC cells activated PDGFR tyrosine phosphorylation, but only AKT activation was PDGFR-dependent. Furthermore, we found that connective tissue growth factor (CTGF), a member of the CCN family, was the most pronouncedly induced CAFs effector gene by GC cells. Knockdown of CTGF impaired the ability of CAFs to promote GC cell migration and invasion. Although the PDGFR-AKT pathway was pronouncedly activated in CAFs stimulated by GC cells, its pharmacological inhibition affected neither CTGF induction nor CAFs-induced GC cell migration. Unexpectedly, the knockdown of SRC and SRC-family kinase inhibitors, dasatinib and saracatinib, significantly impaired CTGF induction in activated CAFs and the migration of GC cells co-cultured with CAFs. SRC inhibitors restored the reduced expression of epithelial markers, E-cadherin and Zonula Occludens-1 (ZO-1), in GC cells co-cultured with CAFs, as well as CAFs-induced aggregate formation in a 3D tumor spheroid model.

**Conclusions:**

This study provides a characterization of the signaling pathways and effector genes involved in CAFs activation, and strategies that could effectively inhibit it in the context of GC.

Video Abstract

**Supplementary Information:**

The online version contains supplementary material available at 10.1186/s12964-023-01396-7.

## Introduction

Gastric cancer (GC) is a common malignant tumor with high incidence and mortality worldwide [1]. Although perioperative chemotherapy has markedly improved the prognosis of patients with advanced GC, the survival of the most patients remains limited [2]. Treatment with trastuzumab, a humanized monoclonal antibody targeting HER2, has shown a favorable prognosis for patients with HER2-positive advanced GC [3, 4]. However, HER2-amplified patients account for only 10–25% of all GC patients [5], highlighting the need for novel strategies to overcome advanced GC.

The tumor stromal cells in the tumor microenvironment (TME), consisting of fibroblasts, endothelial cells, immune cells, and extracellular matrix, contribute to cancer development [6]. Cancer-associated fibroblasts (CAFs), a major component of TME, play an important role in tumor development and drug resistance by secreting various molecules, including cytokines, chemokines, and growth factors [7, 8]. Several studies have reported that CAFs-derived molecules enhance migration, invasion, and chemotherapy resistance in GC cells [9, 10]. A CAFs-specific gene signature has been identified to predict pathological characteristics, cancer stem cell index, drug sensitivity, immune-related signature, and prognosis of patients with GC [11], indicating that therapeutic approaches targeting CAFs can be an effective strategy for overcoming GC.

Connective tissue growth factor (CTGF), also known as CCN2, has four conserved domains: an insulin-like growth factor binding protein-like module (IGFBP), a von Willebrand factor type C repeat module (VWC), a thrombospondin type-1 repeat module (TSP-1), and a cysteine-knot-containing module (CT). The multi-modular structure of CTGF may exert biological functions through interaction with other proteins [12]. Overexpression of CTGF has been found in various cancers, including pancreatic cancer, prostate cancers, and gliomas, acute lymphoblastic leukemias, esophageal squamous cell carcinomas [13–17], and is implicated in tumor progression by regulating proliferation, migration, invasion, and epithelial–mesenchymal transition (EMT) of cancer cells [18]. In the context of GC, CTGF is significantly upregulated in GC tissues and played an important role in GC cell growth and invasion [19, 20], and its elevated expression is associated with a poor prognosis of GC patients [21]. Although CTGF expression is known to be induced by multiple stimuli, including transforming growth factor-β (TGF-β) signaling, angiotensin II, thrombin, hypoxia, and mechanical stress [18], the causes of aberrant CTGF expression in tumor tissues of GC patients have not been clearly revealed. Previous studies have focused on CTGF function in GC cells [19, 20], but the molecular mechanism by which CTGF is regulated and its role in gastric CAFs remains to be elucidated.

It is increasingly being recognized that inhibiting cancer-associated fibroblasts (CAFs) can have several beneficial effects on cancer therapy outcomes. To develop effective therapeutic strategies targeting CAFs, it is important to understand the signaling pathways involved in CAFs activation. In this study, we found that GC-stimulated CAFs activated the PDGFR-dependent AKT pathway and the independent SRC pathway. We also found that GC cells upregulated CTGF in CAFs, and knockdown of CTGF impaired the ability of CAFs to promote GC cell migration and invasion. Knocking down SRC and SRC-family kinase (SFK) inhibitors, dasatinib and saracatinib, significantly impaired the induction of CTGF in activated CAFs. SFK inhibitors hindered the migration of GC cells induced by CAFs and also the reduced expression of epithelial markers, E-cadherin and Zonula Occludens-1 (ZO-1), in GC cells co-cultured with CAFs. Finally, dasatinib significantly disrupted the formation of CAFs-induced aggregates in a 3D tumor spheroid model. Collectively, our study provides novel insights into signaling pathways involved in CAFs activation and potential therapeutic strategies to inhibit CAFs function in the context of GC.

## Materials and methods

### Cell culture

Normal gastric-associated fibroblasts (NAFs) and gastric cancer-associated fibroblasts (CAFs) were isolated from GC specimens as described previously [22]. The normal gastric epithelial cell line (HFE145) and MKN28 cells were obtained from the American Type Culture Collection (ATCC), and all other GC cell lines (SNU216, SNU484, SNU601, SNU638, SNU668, MKN1, MKN45, MKN74, AGS) from the Korean Cell Line Bank. Fibroblasts and HFE145 cells were cultured in DMEM containing 10% FBS and 1% antibiotic-antimycotic (HyClone, Logan, UT, USA). All GC cell lines were cultured in RPMI1640 with 10% FBS and 1% antibiotic-antimycotic (HyClone, Logan, UT, USA). All cells were incubated at 37 °C in a humid environment with 5% CO_2_.

### Chemicals and antibodies

All kinase inhibitors were purchased from Selleckchem (Houston, TX, USA) and dissolved in dimethyl sulfoxide (DMSO). Primary antibodies, except for β-actin (which was purchased from Santa Cruz Biotechnology), were obtained from Cell Signaling Technology (Danvers, MA, USA). Horseradish peroxidase (HRP)-conjugated secondary antibodies were purchased from Thermo Fisher Scientific (Waltham, MA, USA).

### Conditioned media (CM) collection

1.8 × 10^6^ cells were seeded in 10 cm dishes for 60–70% confluency. After overnight attachment and growth, cells were washed with PBS and cultured in serum-free media. After 48 h, media were centrifuged at 1200 rpm for 3 min to remove debris.

### Western blotting

Cells were washed with ice-cold PBS, and lysed in NETN lysis buffer (100 mM NaCl, 20 mM Tris pH 8.0, 0.5 mM EDTA, 0.5% NP-40) supplemented with protease and phosphatase inhibitor cocktail (GenDepot, Baker, TX, USA). Whole cell extracts were resolved on SDS-PAGE and transferred to nitrocellulose membrane. The membrane was blocked with 5% skim milk in TBST and incubated with primary antibodies at 4 °C overnight. After washing three times with TBST, the membrane was incubated with horseradish peroxidase (HRP)-conjugated secondary antibodies at room temperature for 1 h. The Li-cor system (LI-COR Biosciences, Lincoln, NE, USA) was used to detect chemiluminescence.

### Phospho-RTK array

Tyrosine phosphorylation of receptor tyrosine kinases was analyzed using Human phospho-receptor tyrosine kinase array kit (R&D systems, Minneapolis, MN, USA) according to the manufacturer’s protocol. Briefly, 80–90% confluent cells were lysed in the lysis buffer provided by the kit. The array membrane was blocked with array buffer 1, loaded with 300 μg of lysates, and then incubated at 4 °C overnight. After washing, the membrane was incubated with HRP-conjugated anti-phospho-tyrosine antibodies at room temperature for 2 h. The chemiluminescence was detected as described above.

### qPCR

Total RNA was extracted using RNA Extraction Kit (Bioneer, Daejeon, South Korea) according to the manufacturer’s instructions. The cDNA was synthesized using the CellScript™ cDNA Master Mix (CellSafe, Yongin, South Korea). The mixture with cDNA, SYBR Green (Toyobo, Osaka, Japan), forward primer, reverse primer was subjected to 35 cycles of PCR amplification using the following cycling conditions: denaturation at 95 °C for 5 s, annealing at 55 °C for 10 s, and extension at 72 °C for 30 s. The expression level of each mRNA was normalized to that of GAPDH. All primer sequences used in this study are listed in supplementary Table 1.

### ELISA

Quantification of CTGF was performed using Human CTGF ELISA Kit (R&D System, Minneapolis, MN, USA) according to manufacturer’s instructions. Briefly, the plate was coated with capture antibodies, and the samples were added and incubated for 2 h at room temperature. After washing samples, detection antibodies were added for 2 h. Then, working dilution of Streptavidin-HRP was added. After incubation at room temperature for 20 min in the dark, the reaction was stopped by adding 50 μl of stop solution. The plate was read at 450 nm via a microplate reader.

### siRNA transfection

siRNA transfections were performed using RNAiMAX reagent (Thermo Fisher Scientific, Carlsbad, CA, USA) according to the manufacturer’s instructions. siRNAs were purchased from Genolution (Seoul, South Korea). The siRNA duplex sequences used in this study are as follows: siControl: sense:5′-CCUCGUGCCGUUCCAUCAGGUAGUU-3′; antisense:5′-CUACCUGAUGGAACGGCACGAGGUU-3′, siCTGF #1: sense:5′-CUGUACUACAGGAAGAUGUUU-3′, antisense:5′-ACAUCUUCCUGUAGUACAGUU-3′, siCTGF #2: sense:5′-CAACUGUCCCGGAGACAAUUU-3′, antisense: 5′-AUUGUCUCCGGGACAGUUGUU-3′, siSRC #1: sense: 5′- CCACCUUUGUGGCCCUCUAUU-3′, antisense: 5′-UAGAGGGCCACAAAGGUGGUU-3′, siSRC #2: sense: 5′-GCAAUCAAGCAGACAUAGAUU-3′, antisense: 5′-UCUAUGUCUGCUUGAUUGCUU-3′.

### Invasion/migration assay

CAFs were seeded at a density of 5 × 10^4^ cells in the bottom chamber. On the following day, trypsinized GC cells were washed twice with PBS, suspended in serum-free medium, and then added to the upper chamber membrane (8.0 μm pore size) of a transwell chamber (65 mm Costar Transwell chamber, Corning, New York, NY, USA). After 24 h of incubation, non-migrating cells in the chamber were removed using a cotton swab, and the migrated cells were fixed with 4% paraformaldehyde. Subsequently, cells were stained with 0.5% crystal violet for 20 min. Migrated cells were observed and counted from three randomly chosen fields using ImageJ software under a phase-contrast microscope. For the invasion assay, the transwell filter was coated with Matrigel, and cells were incubated for 48 h before quantification of the invaded cells.

### 3D tumor spheroid model

3D spheroids were generated as described previously [23, 24]. Briefly, 4 × 10^3^ of GC or CAF cells suspended in culture medium were loaded into each well of 96-well round-bottom ultra-low attachment microplates (Corning, New York, NY, USA). For the formation of bicellular GC/CAFs spheroids tumor spheroids, 4 × 10^3^ of GC and CAF cells were mixed at a 1:1 ratio, then incubated as described above. Spheroid size was determined by measuring the longest and shortest diameters of each spheroid using ImageJ software (http://imagej.nih.gov/ij/).

## Result

### GC cells stimulate PDGFR-dependent and -independent pathways in CAFs

**Fig. 1:**
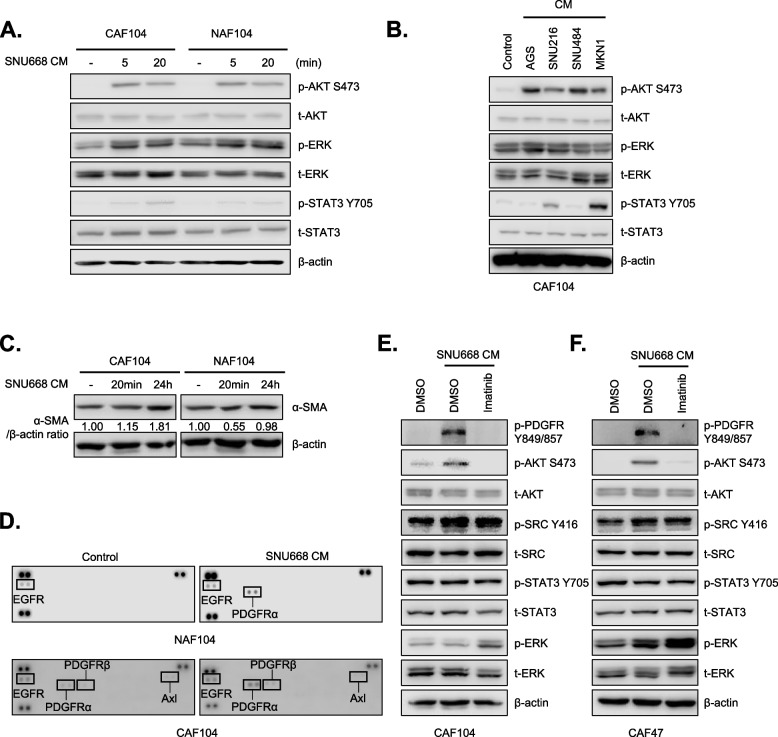
CM derived from GC cells stimulate multiple signal transduction pathways in CAFs. **A**. Normal-associated fibroblasts (NAFs) or cancer-associated fibroblasts (CAFs) were treated with conditioned media (CM) obtained from SNU668 cells for 5 and 20 min. The activities of STAT3 (79/86 KDa), AKT (60 KDa), and ERK (42/44 KDa) were analyzed by western blotting, with β-actin (45 KDa) serving as an internal control. **B**. CAFs were treated with CM obtained from different gastric cancer (GC) cells for 20 min, and the activities of STAT3, AKT, and ERK were analyzed by western blotting. **C**. NAFs or CAFs were treated with SNU668 CM for 20 min or 24 h, and the level of α-SMA (42 KDa) was analyzed by western blotting. **D**. NAFs or CAFs were treated with SNU668 CM for 5 min, and the tyrosine phosphorylation levels of receptor tyrosine kinases (RTKs) were analyzed by phospho-RTK arrays. **E** and **F**. Two different CAFs (CAF104 and CAF47-hTERT) were treated with SNU668 CM along with 5 μM of imatinib for 6 h, and the activities of PDGFR (190 KDa), AKT, ERK, SRC (60 KDa), and STAT3 were analyzed by western blotting

To investigate how GC cells modulate cancer-related signal transduction pathways in neighboring fibroblasts, CAFs or NAFs were treated with conditioned media (CM) obtained from a GC cell line, SNU668. Western blot analysis showed that SNU668 CM pronouncedly upregulated AKT phosphorylation, while it marginally induced ERK and STAT3 phosphorylation (Fig. 1A). We extended this observation to CM from other panels of GC cell lines. AKT phosphorylation was significantly induced by all CM we tested, and only SNU216 and MKN1 CM could induce STAT3 phosphorylation. Changes in ERK phosphorylation was marginal presumably due to basal ERK activity in CAFs (Fig. 1B). We also observed CM from SNU668 could upregulate α-SMA expression, which is an indicative of activated CAFs, in CAFs (Fig. 1C). The upregulated phosphorylations are known to be downstream signaling molecules of receptor tyrosine kinases (RTKs). We thus conducted phospho-RTK arrays to identify upstream RTKs that could be responsible for signaling activation by GC CM. We found that tyrosine phosphorylation of PDGFRα was specifically induced by CM from SNU668 in both NAFs and CAFs (Fig. 1D). Western blot analysis validated tyrosine phosphorylation of PDGFRα/β (Y849/857), linked to tyrosine kinase activity of PDGFRα, was significantly upregulated by SNU668 CM (Fig. 1E). This result was also validated in an additional CAFs model, an immortalized CAFs cell line by stable expression of hTERT (CAF47) [25] (Fig. 1F). SNU668 CM-induced AKT phosphorylation was abolished by imatinib, a tyrosine kinase inhibitor (TKI) against PDGFR, indicating that AKT activation by CM was driven by PDGFRα. In this western blot analysis, we also observed that SNU668 CM could upregulate activating phosphorylation of SRC (Y416), which was marginally affected by imatinib. These results indicate that GC stimulates CAFs through AKT and SRC activation in both PDGFRα-dependent and –independent manners.

### CTGF expression is elevated in CAFs stimulated by GC cells

**Fig. 2:**
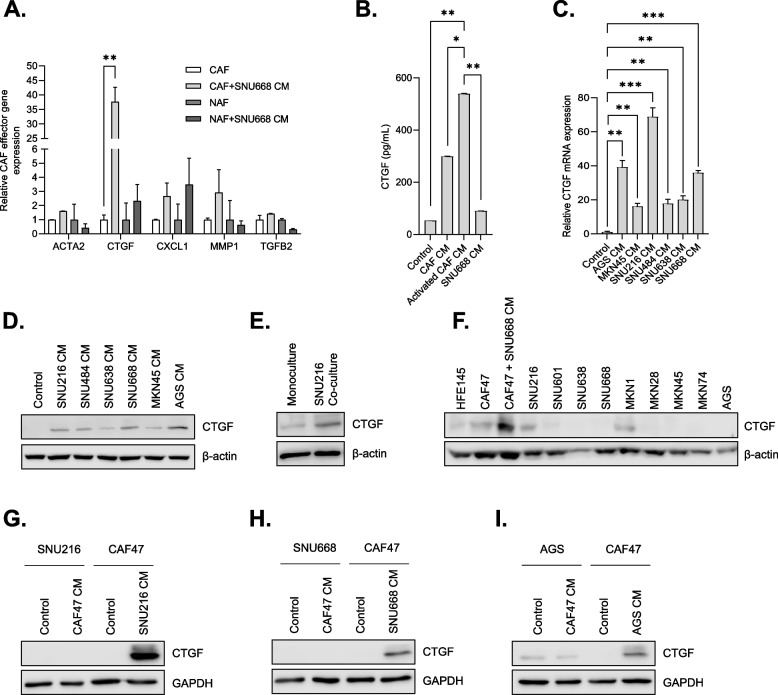
Induction of CTGF in CAFs stimulated by GC CM, with limited expression in GC cells. **A**. NAFs or CAFs were treated with CM obtained from SNU668 cells for 6 h. Quantitative PCR (qPCR) was used to analyze the mRNA levels of CAF effector genes. **B**. The concentration of CTGF (connective tissue growth factor) protein in serum-free media (control) or CM obtained from unstimulated CAFs, CAFs stimulated by SNU668 CM, and SNU668 cells was analyzed by enzyme-linked immunosorbent assay (ELISA). **C** and **D**. CAF47 cells were treated with CM obtained from different GC cell lines for 6 h, and the CTGF mRNA levels (C) and protein levels (D) were analyzed by qPCR and western blotting, respectively. **E**. CAF47 cells were co-cultured with SNU216 cells for 24 h, and CTGF (35 KDa) expression was analyzed by western blotting. **F**. CTGF expression was analyzed by western blotting in various cell lines: GC cell lines, the normal gastric epithelial cell line (HFE145), and CAFs (both untreated and stimulated with SNU668 CM for 6 h). G-I. CTGF expression was assessed by western blotting in SNU216 (G), SNU668 (H), and AGS (I) cells stimulated by CAF47 CM for 6 h. As a positive control, CAFs lysate stimulated by GC CM was used. Error bars indicate the standard deviation of representative triplicates from at least three experiments, which showed similar results. NS: not significant, *: *p* < 0.05, **: *p* < 0.01, ***: *p* < 0.001

CAFs-derived factors, including growth factors and pro-migratory cytokines, enhance the metastatic potential of cancer cells leading to cancer progression [8]. Activation of RTK-driven signaling pathways by GC cells could lead to enhanced expression of secretory factors in CAFs, which promote their tumor-promoting function [26, 27]. To test this possibility, CAFs were treated with SNU668 CM and examined for the expression of genes implicated in CAFs activation [26]. ACTA2, also known as α-smooth muscle actin (α-SMA), is a key characteristic of CAFs, whose high stromal expression was associated with enhanced angiogenesis, tumor growth, lymph node metastasis, frequency of cancer stem cells and worse clinical outcome [28–32]. TGF-β is known as a prominent CAFs-derived growth factor, which contributes to cancer migration, and invasion by inducing epithelial-mesenchymal transition (EMT) [33]. It can also facilitate the expression of fibrotic factors such as connective tissue growth factor (CTGF) [34]. CXCL1, a chemokine expressed in tumor cells or stromal cells, was reported to mediate angiogenesis and promote tumor progression [35]. Among these prominent factors, we found that SNU668 CM significantly upregulated the expression of CTGF (Fig. 2A). Unlike CAFs, we didn’t observe a noticeable increase of CTGF expression in NAFs. The increased secretion of CTGF by GC CM was confirmed by ELISA experiments (Fig. 2B). We extended this observation to CM from other panels of GC cell lines. CTGF mRNA and protein expression was significantly induced by all GC CM we tested (Fig. 2C and D). We also observed that CTGF expression in CAFs was elevated when co-cultured with SNU216 cells (Fig. 2E). The CTGF expression induced by GC was detected in CAFs lysate after 6 h of CM treatment and barely detectable after 12 h of CM treatment (Supplementary Fig. 1). Next, we gauged the correlation between the expression of CTGF and known CAF markers in gastric cancer tissues, employing public databases. The Cancer Genome Atlas (TCGA) database showed that CTGF expression was highly correlated with the expression of ACTA2 (*r* = 0.66, *P* = 4.7e-53) and FAP (*r* = 0.61, *P* = 8.6e-44), both of which are considered as markers of CAFs [36] (Supplementary Fig. 2A and B). Its expression was also significantly associated with the poor prognosis of GC patients (Supplementary Fig. 2C). To emphasize the significance of stromal expression of CTGF in GC progression, we assessed CTGF expression across a panel of GC cell lines (*n* = 9) and a normal gastric epithelial cell line (HFE145). CTGF expression was generally found to be negligible in GC cell lysates when compared to CAFs stimulated by GC CM (Fig. 2F and Supplementary Fig. 3A). We also explored the possibility that CM derived from CAFs could promote CTGF expression in GC cells. To examine this, we stimulated three GC cell lines (SNU216, SNU668, AGS) with CAF CM and assessed CTGF expression. The expression of CTGF in GC cells stimulated by CAF CM was barely detectable compared to those in CAFs stimulated by GC CM (Fig. 2G–I and Supplementary Fig. 3B). Collectively, these findings indicate that CTGF derived from CAFs stimulated by GC cells might serve as a crucial mediator of tumorigenesis in the context of GC.

### CTGF is important for CAFs-induced GC cell migration and invasion

**Fig. 3:**
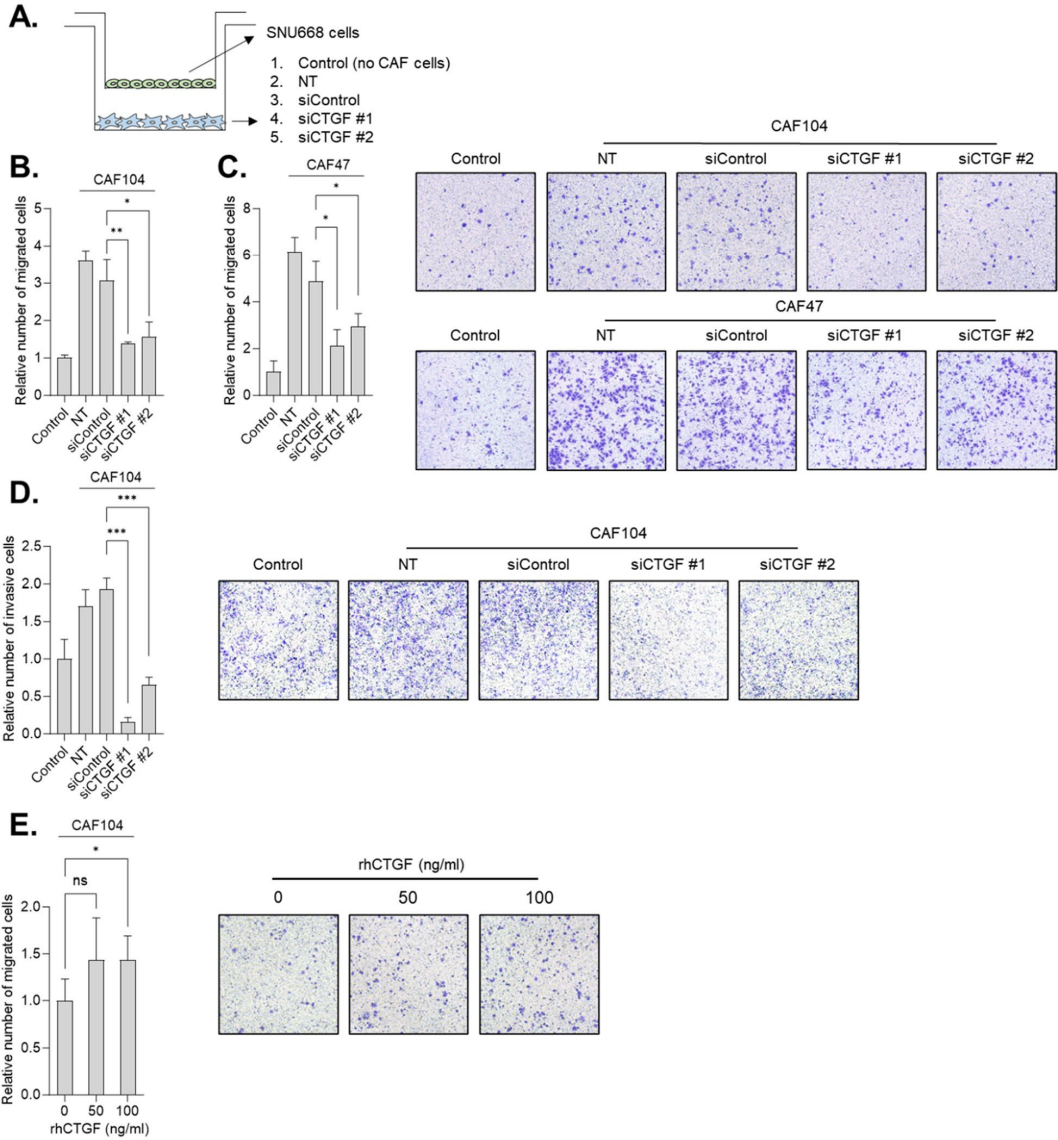
CTGF plays an important role in CAFs to promote GC cell migration and invasion. **A**. A schematic diagram of a transwell migration/invasion assay in a co-culture system. **B** and **C**. The migration of SNU668 cells co-cultured with siRNA-transfected CAF104 (B) and CAF47 (C) was analyzed by transwell migration assay. The knockdown efficiency of CTGF was validated by qPCR and western blotting (Supplementary Fig. 4A–D) **D**. The invasion of SNU668 cells co-cultured with siRNA-transfected CAF104 was analyzed by transwell invasion assay. **E**. The migration of SNU668 cells incubated with different concentrations of CTGF was analyzed by transwell migration assay. Representative images of migrated or invaded cells on the membrane (magnification, 200x) are shown. Error bars indicate the standard deviation of representative triplicates from at least three experiments, which showed similar results. NT : non-transfected. NS: not significant, *: *p* < 0.05, **: *p* < 0.01, ***: *p* < 0.001

It has been reported that CTGF promotes GC cell growth and metastasis [19, 20] and is an independent predictor of poor prognosis in GC patients [37]. These studies were focused on CTGF function expressed in GC cells. To investigate the importance of CTGF in GC-activated CAFs, we silenced CTGF in CAFs and tested whether it could impair GC migration driven by CAFs (Fig. 3A and Supplementary Fig. 4A–D). The transwell migration assay showed that silencing CTGF inhibited the ability of CAFs to enhance GC cell migration in two independent CAFs cell line models (Fig. 3B and C). We also observed that silencing CTGF inhibited the invasion of GC cells promoted by co-culture with CAFs (Fig. 3D). Consistent with previous results, recombinant human CTGF (rhCTGF) enhanced GC migration (Fig. 3E). It is possible that CAFs-derived CTGF could promote the proliferation of GC cells rather than enhancing their ability to migrate and invade. To exclude this possibility, we silenced CTGF in a GC cell line, SNU484, whose CTGF expression was the most pronounced (Supplementary Fig. 3A). We then examined whether knocking down CTGF could affect the viability of these cells. The MTT assay showed that silencing CTGF failed to inhibit the proliferation of SNU484 cells (Supplementary Fig. 5). Taken together, these results indicate that CTGF derived from GC-activated CAFs plays an important role in promoting cancer cell migration and invasion.

It has been reported that CTGF can activate the AKT pathway [38, 39]. Therefore, we investigated whether CAFs-derived CTGF is responsible for the signaling activation induced by GC CM and whether CTGF could affect CAF proliferation in an autocrine manner. Our findings revealed that CTGF knockdown did not impair the activation of signaling proteins in CAFs stimulated by CM obtained from SNU668 cells, nor did it affect the viability of CAFs (Supplementary Figs. 6 and 7). In line with these results, recombinant human CTGF (rhCTGF) failed to increase CAF proliferation (Supplementary Fig. 8).

### SRC inhibition impaired CTGF expression in activated CAFs

**Fig. 4:**
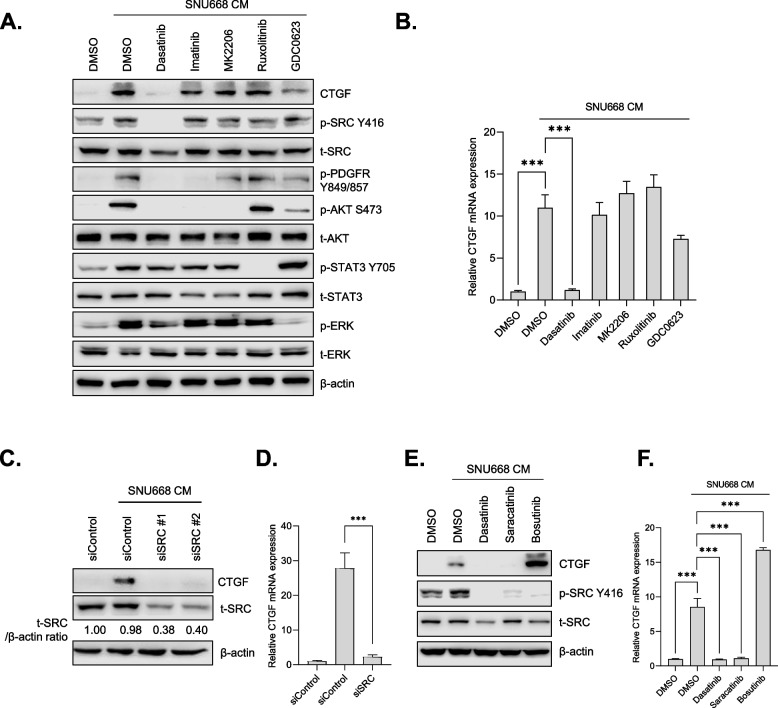
Dasatinib and saracatinib decrease GC-induced CTGF expression in CAFs. **A** and **B**. CAF47 cells were treated with SNU668 CM along with different kinase inhibitors (5 μM of dasatinib, 5 μM of imatinib, 1 μM of MK2206, 1 μM of ruxolitinib, 1 μM of GDC0623) for 6 h, the expression of CTGF (35 KDa), SRC (60 KDa), PDGFR (190 KDa), AKT (60 KDa), STAT3 (79,86 KDa), ERK (42/44 KDa), β-actin (45 KDa) was analyzed by western blotting (A) and qPCR (B). **C** and **D**. CAF47 cells were transfected with siSRC, then stimulated with SNU668 CM for 6 h, then the expression of CTGF, SRC was analyzed by western blotting (C) and qPCR (D). **E** and **F**. CAF47 cells were treated with SNU668 CM along with SRC-family kinase (SFK) inhibitors (500 nM of dasatinib, 500 nM of saracatinib, 500 nM of bosutinib) for 6 h, and the expression of CTGF, SRC, β-actin was analyzed by western blotting (E) and qPCR (F). Error bars indicate the standard deviation of representative triplicates from at least three experiments, which showed similar results. NS: not significant, *: *p* < 0.05, **: *p* < 0.01, ***: *p* < 0.001

Next, we attempted to identify the signaling pathways responsible for the elevated expression of CTGF in GC-activated CAFs. We reasoned that the identification of kinase inhibitors that could effectively inhibit CTGF expression could provide useful information on the mechanism involving the regulation of CTGF expression, as well as therapeutic insights into targeting CAFs in GC therapy. To this aim, we treated SNU668CM with a panel of kinase inhibitors in CAFs and then examined CTGF expression. Although SNU668 CM significantly upregulated the PDGFR-AKT pathway, inhibitors targeting this pathway, imatinib (PDGFR inhibitor) and MK2206 (AKT inhibitor), failed to efficiently inhibit CM-induced CTGF expression. Notably, dasatinib, a known multi-target kinase inhibitor targeting BCL-ABL and SRC family kinases, abolished CTGF expression (Fig. 4A and Supplementary Fig. 9). These results were verified by RT-qPCR analyses, which showed that SNU668 CM-induced CTGF mRNA expression was significantly impaired by dasatinib (Fig. 4B). We compared the effect of dasatinib on CTGF expression to that of a panel of TKIs, including lapatinib, erlotinib, sorafenib, and crizotinib, which have been investigated in clinical studies for GC treatment [40–43]. Consistent with the findings shown in Fig. 4B, only dasatinib demonstrated a significant inhibitory effect on CTGF mRNA induction in the activated CAFs (Supplementary Fig. 10). The inhibitory effect of dasatinib on CTGF expression was also observed at nano-molar range (Supplementary Fig. 11; 50 nM to 1000 nM). While SFKs are the main targets of dasatinib in solid cancers, studies have reported a broad target spectrum of dasatinib [44, 45]. We previously reported the target profile of dasatinib in the context of GC [46]. To determine the involvement of SRC in CTGF expression within activated CAFs, we investigated whether silencing SRC expression using siRNA could hinder GC-induced CTGF expression. The suppression of SRC expression in CAFs significantly impaired both the protein and mRNA levels of CTGF when treated with CM from SNU668 cells (Fig. 4C and D). Moreover, we observed enhanced CTGF expression in activated CAFs compared to NAFs (Fig. 2A). This finding correlated with the more pronounced induction of SRC phosphorylation by GC CM in CAFs, compared to NAFs (Supplementary Fig. 12), underscoring the pivotal role of SRC activation in controlling CTGF expression in CAFs. We proceeded to investigate if other SFK inhibitors exhibited similar effects. The SRC inhibitor, saracatinib, effectively inhibited SNU668 CM-induced CTGF expression similar to dasatinib (Fig. 4E and F, and Supplementary Fig. 13). Unexpectedly, the other SRC inhibitor, bosutinib, significantly elevated CTGF expression (Fig. 4E and F). This could be attributed to our observation that bosutinib treatment triggered compensatory activation of signaling pathways, involving MEK-ERK signaling (Supplementary Fig. 14). Despite this, co-administration of bosutinib and a MEK inhibitor failed to entirely block CTGF expression (Supplementary Figs. 14, 15A and B). This indicates the potential for feedback activation of multiple signaling pathways, extending beyond the MEK-ERK pathway, leading to enhanced CTGF expression after bosutinib treatment. In summary, our findings suggest that SRC activation is essential for GC CM-induced CTGF expression, and specific SRC inhibitors (dasatinib and saracatinib) hold promise for suppressing CTGF expression in CAFs.

### SRC inhibitors inhibit CAFs-induced GC cell migration as well as aggregate compaction in 3D tumor spheroid model

**Fig. 5:**
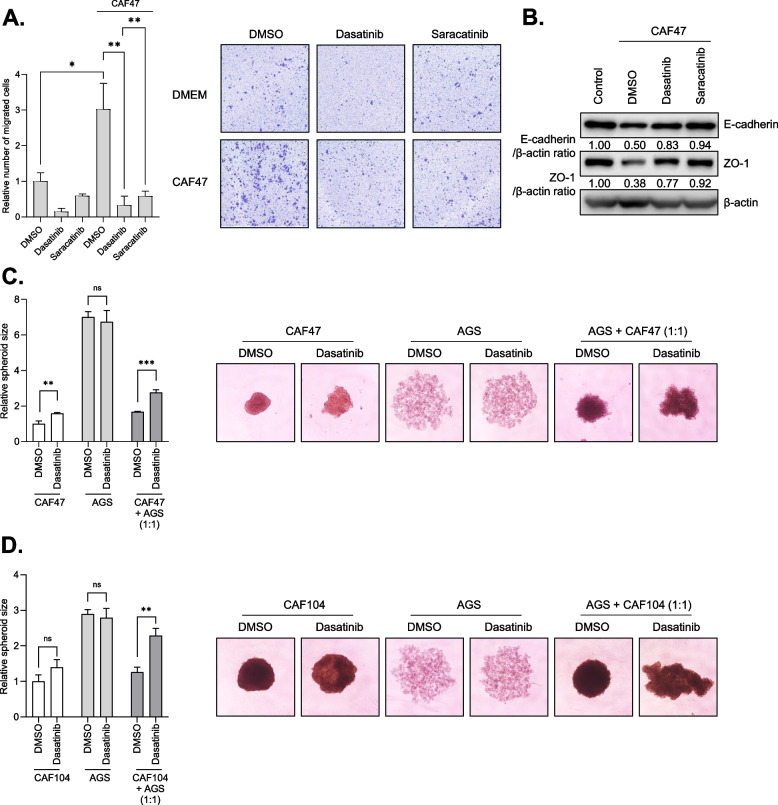
Dasatinib and saracatinib inhibit migration and tumor aggregate formation of GC cells promoted by activated CAFs. **A**. The migration of SNU668 cells co-cultured with drug-treated CAF47 cells was analyzed by transwell migration assay. **B**. SNU668 cells were co-cultured with drug-treated CAF47 cells for 48 h, and E-cadherin (135 KDa) and ZO-1 (220 KDa) expression was analyzed by western blotting. **C** and **D**. *mono*- or bicellular 3D spheroids were generated using AGS cells with CAF47 (C) or CAF104 (D) cells. Dasatinib was treated at a concentration of 1 μM. Error bars indicate the standard deviation of representative triplicates from at least three experiments, which showed similar results. NS: not significant, *: *p* < 0.05, **: *p* < 0.01, ***: *p* < 0.001

Next, we tested if SFK inhibitors which could inhibit CTGF induction in CAFs could inhibit the ability of CAFs to promote GC progression. We found that dasatinib and saracatinib inhibited the migration of GC cells induced by co-culture with CAFs (Fig. 5A), while PDGFR and AKT inhibitors that could not inhibit CTGF induction showed marginal effects on the migration of GC cells (Supplementary Fig. 16). Similarly, TKIs that have been utilized in clinical studies for GC treatment, including lapatinib, erlotinib, sorafenib, and crizotinib, did not significantly impair GC cell migration promoted by CAFs (Supplementary Fig. 17). Previous studies have shown that various microRNA targeting CTGF enhanced the expression of E-cadherin in diverse cancers including colorectal cancer, hepatocellular carcinoma, esophageal squamous cell carcinoma [47–49]. In the context of GC, CTGF reportedly promoted GC cell migration via downregulation of E-cadherin [19]. These previous studies promoted us to test whether CTGF-targeting drugs, dasatinib and saracatinib, could inhibit the ability of CTGF to reduce E-cadherin. We co-cultured SNU668 cells with CAFs exposed to these drugs, and then analyzed the expression of E-cadherin along with another epithelial marker, ZO-1 [50]. We found that the expression of these epithelial markers in SNU668 cells was downregulated by co-culture with CAFs, and dasatinib- or saracatinib-treated CAFs failed to impair their expression in SNU668 cells (Fig. 5B).

While two-dimensional (2D) culture-based models have been extensively used in cancer research and preclinical drug discovery endeavors, they frequently overlook tumor complexity and physiological relevance. This limitation restricts the precise prediction of in vivo efficacy [51]. While mouse models have been extensively used as in vivo models, the high cost and time needed for these experiments have impeded their widespread implementation. There has been a growing recognition in three-dimensional (3D) tumor spheroid model because it can better recapitulate in vivo tumor complexity compared to 2D-based models [52]. Multicellular tumor spheroids (MCTS), consisting of one or more cell types, are recognized as a prominent and extensively investigated model in preclinical oncology [53]. Their inherent ability to replicate critical aspects of real tumors has rendered 3D MCTS an invaluable tool for evaluating the effectiveness of diverse therapeutic interventions. Consequently, MCTS has demonstrated a compelling potential to predict the in vivo efficacy of distinct chemotherapeutic agents, with treatment responses in the MCTS model closely resembling in vivo conditions [54, 55]. Especially, bicellular spheroids model composed of cancer cells and CAFs has proven its utility to investigate reciprocal stromal-epithelial interactions at more physiologically relevant settings in the context of gastric cancer [54, 56]. To validate our findings in a setting closer to an in vivo environment, we generated bicellular tumor spheroids using a panel of GC cells and CAFs. Notably, we observed that bicellular GC/CAFs spheroids were more densely packed in comparison to monocellular GC spheroids. This consistently aligns with a previous study that demonstrated a link between high spheroid compactness and poor differentiation, reduced expression of E-cadherin in GC cells [57] as well as our observation of reduced E-cadherin expression in GC cells when co-cultured with CAFs (Fig. 5B). Furthermore, our results revealed that dasatinib treatment impaired CAFs-induced spheroid compactness, while its effect was confined in monocellular spheroids (Fig. 5C and D and Supplementary Fig. 18A and B). We also observed that TKIs previously investigated in clinical studies for GC treatment, including lapatinib, erlotinib, sorafenib, and crizotinib, did not significantly reduce CAFs-induced spheroid compactness (Supplementary Fig. 19). This finding is consistent with the results described above (Supplementary Figs. 10 and 17). Correctively, these results suggest that dasatinib could potentially suppress the activity of CAFs that promote spheroid compactness, possibly through the inhibition of secretory molecules’ expression including CTGF.

## Discussion

The latest progress in comprehending the molecular mechanisms underlying GC has provided hope that targeted therapies can be leveraged to improve survival and reduce toxicity. Therefore, targeted therapies are being attempted for the treatment of GC [58, 59]. Trastuzumab, also known as Herceptin, is a representative molecular targeted therapy for GC in HER2-positive patients; however, the emergence of drug resistance remains a major hurdle. Comprehensive molecular characterization of gastric adenocarcinomas has resulted in the identification of potential molecular targets for GC treatment [60]. CAFs-derived molecules have been reported to be involved in metastasis, angiogenesis, and resistance to chemotherapy of GC [9]. A recent study has shown that CAFs mediated trastuzumab resistance in HER2-positive breast cancer [61], suggesting that CAFs could also contribute to tyrosine kinase inhibitor (TKI) resistance in gastric cancer. It is thus important to understand the underlying molecular mechanisms regulating CAFs’ function and identify potential therapeutic targets that inhibit CAFs’ activity to enhance outcomes of GC molecular therapy.

In this study, we characterized receptor tyrosine kinases-driven signaling pathways in CAFs, which are induced by GC cells. We demonstrated that CM derived from GC cells could stimulate PDGFR-dependent AKT pathway. AKT pathway is linked to various molecular mechanism of CAFs activation [62]. The PDGFR activation has been known as one of traditional CAFs biomarkers and linked to tumor promoting functions of CAFs [63]. The stromal PDGFR signaling is associated with poor prognosis of breast cancer [64] and blocking stromal PDGFR activation impaired tumor progression in genetically engineered mouse model of cervical carcinogenesis [65]. In the context of GC, the stromal expression of PDGFR is associated with GC tumor progression [66] and its pharmacological inhibition by imatinib could impair GC progression in mouse model [67]. Although we observed PDGFR was the only activated RTK in CAFs stimulated by GC cells, the inhibition of PDGFR and PDGFR-driven AKT activation by imatinib and MK2206 failed to impair GC-driven CTGF expression in CAFs (Fig. 4A and B) as well as the function of CAFs to promote GC cell migration (Supplementary Fig. 16). These results suggest that blocking single RTK-driven signaling pathway could not be sufficient to block CAFs function, while multi-target kinase inhibitors such as dasatinib could be more effective for stromal targeting in GC.

What is the mechanism of SRC activation in CAFs activated by GC cells? Several cytokines secreted by SNU668, including CCL2, CXCL1, IL-6, IL-8, Mif, and PAI-1, have been shown to play a significant role in SRC activation [10]. For instance, CCL2 has been reported to promote proliferation and cell cycle progression by activating SRC and PKC in basal-like breast cancer cell lines [68]. Similarly, the CXCL1-LCN2 axis has been linked to the induction of epithelial-mesenchymal transition (EMT) and SRC signaling activation in prostate cancer cells [69]. IL-6 and IL-8 have been associated with the promotion of GC invasion through the activation of the SRC signaling pathway [70, 71]. Furthermore, the expression of GC-derived TGF-β1 has shown a significant correlation with the malignancy grade [72, 73]. Like the other GC-derived molecules mentioned earlier, TGF-β1 has also been reported to induce SRC phosphorylation in various cell types [74–76]. Notably, TGF-β1-induced SRC signaling leads to enhanced CTGF expression. Inhibiting SRC with saracatinib has been shown to attenuate TGFβ1-induced CTGF expression in AML12 cells, primary hepatocytes, and LX2 cells [74]. Inhibiting SRC activated by TGF-β1 impaired the activation of ERK, Smad2, and Smad3, as well as Smad nuclear translocation, which, in turn, inhibited the induction of CTGF in rat osteosarcoma osteoblast-like cells [75]. SRC inhibitors have also been found to reduce the activation of JNK and Smad3 and the induction of CTGF by TGFβ1 stimulation in human gingival fibroblasts [76]. Moreover, Smad3 inhibitors showed a similar effect on reducing CTGF expression induced by TGF-β, comparable to SRC inhibitors [76]. Considering these accumulated results, further research is needed to fully elucidate the signaling pathways involved in SRC activation and its downstream signaling that promotes CTGF expression in CAFs activated by GC. Such research could provide valuable insights into the mechanisms underlying the interaction between GC cells and CAFs.

Our findings also demonstrate that these drugs targeting CTGF could not only inhibit the function of CAFs in promoting GC migration, but they could also reverse the inhibitory effects of co-culturing with CAFs on the expression of E-cadherin and ZO-1 in GC cells (Fig. 5A and B). E-cadherin and ZO-1 play crucial roles in the formation of cell-cell junctions, particularly adherens junctions (AJs) and tight junctions (TJs), which are associated with limited migration ability [77]. While we acknowledge that it is premature to conclusively attribute the effect of these SFK inhibitors solely to their inhibitory role on CTGF expression, as they might modulate a broader panel of secretory genes, we propose that targeting CTGF and its associated signaling pathways in CAFs shows promise as a therapeutic strategy. Such targeting could inhibit the tumor-promoting functions of CAFs and potentially enhance the outcomes of molecular therapy for gastric cancer.

Our study focused on the role of CAFs-derived CTGF in the context of epithelial and stromal crosstalk. The role of CTGF in the tumor microenvironment (TME) has been extensively investigated, primarily focusing on its impact on cancer cell phenotypes, encompassing proliferation [78, 79] migration/invasion [19, 80] and EMT [81, 82]. Nevertheless, it is important to note that CTGF also exerts a significant influence on various components within the TME. CTGF has been identified as an enhancer of vascular endothelial growth factor (VEGF) and angiopoietin 2 expression - essential factors for tumor angiogenesis - thus facilitating tumor growth and metastasis [83, 84]. In vivo studies in murine models demonstrated that CTGF induces the recruitment of inflammatory cells, including T lymphocytes and monocytes/macrophages, through activating NF-κB signaling [85]. These investigations collectively suggest that CAFs-derived CTGF could potentially modulate inflammatory responses and tumor immunity within the TME.

Despite the insights gained from our study into the signal transduction pathways associated with CAFs activation, we acknowledge several limitations in our approach. The MCTS experimental model, where our key findings are validated, has shown compelling potential in predicting the in vivo efficacy of distinct chemotherapeutic agents. However, it’s important to recognize that the complexities of drug absorption, metabolism, efficacy, and toxicity simply cannot be adequately assessed using in vitro techniques alone. Therefore, a valuable future direction would be to investigate the validity of dasatinib as a novel therapeutic approach to inhibit CAFs activation using an appropriate in vivo animal model [54, 55]. Antibody-based receptor tyrosine kinase (RTK) arrays typically utilize predefined sets of antibodies, which might not cover the entire spectrum of signaling molecules involved in CAFs activation. This can result in a biased view of the signaling pathways and potentially overlook critical players that are not included in the array. To address the limitations of antibody-based arrays, future studies could consider adopting a liquid chromatography-mass spectrometry (LC-MS)-based proteomics approach. LC-MS allows for unbiased and quantitative analysis of the entire proteome, providing a comprehensive overview of signaling molecules without relying on predefined antibodies. Prior studies have employed LC-MS-based proteomic profiling to unravel the underlying molecular mechanisms of CAFs activation in diverse cancer contexts [86–88]. The LC-MS based secretome analysis revealed potential contributors to gastric CAFs activation under hypoxic stress [25]. We propose that such a systems-level approach to dissecting the signal transduction pathways involved in GC-activated CAFs could yield novel insights. Furthermore, integration of multi-omics data - such as transcriptomics, proteomics, and metabolomics - can provide a more comprehensive view of the signaling pathways involved in CAFs activation. This integrative approach could help validate findings and identify potential regulatory mechanisms that might be missed using a single approach.

### Supplementary Information

**Additional file 1.**

**Additional file 2.**

## Data Availability

All data generated or analyzed during this study are included in this published article and its supplementary information files.

## Abbreviations

*α*-SMA: *α*-smooth muscle actin
AJs: Adherens junctions
CAFs: Cancer-associated fibroblasts
CM: Conditioned media
CT: Cysteine-knot-containing module
CTGF: Connective tissue growth factor
EMT: Epithelial-mesenchymal transition
GC: Gastric cancer
IGFBP: Insulin-like growth factor binding protein-like module
IF: Intermediate filament
LC-MS: liquid chromatography-mass spectrometry
MCTS: multicellular tumor spheroids
NAFs: Normal-associated fibroblasts
RTK: Receptor tyrosine kinases
TGF-β: Transforming growth factor-β
TJs: Tight junctions
3D: Three-dimensional
TKI: Tyrosine kinase inhibitor
TME: Tumor microenvironment
TSP-1: Thrombospondin type-1 repeat module
2D: Two-dimensional
VEGF: Vascular endothelial growth factor
VWC: Von Willebrand factor type C repeat module
ZO-1: Zonula Occludens-1
