# Supplementary material for: Characterization of gastric cancer-stimulated signaling pathways and function of CTGF in cancer-associated fibroblasts
Source: Cell Commun Signal. 2024 Jan 2;22:8. doi: 10.1186/s12964-023-01396-7 (PMC10763493; doi:10.1186/s12964-023-01396-7)
Supplement: Supplementary file 2 — Additional file 1. [file 12964_2023_1396_MOESM1_ESM.docx]

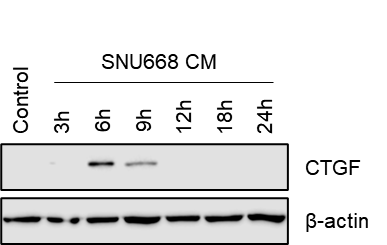


**Supplementary figure 1. CTGF expression at various time points in CAFs stimulated by GC CM.**

CAF47 cells were treated with CM obtained from SNU668 cells at the indicated time points, and CTGF (35 KDa) expression was examined by western blotting.


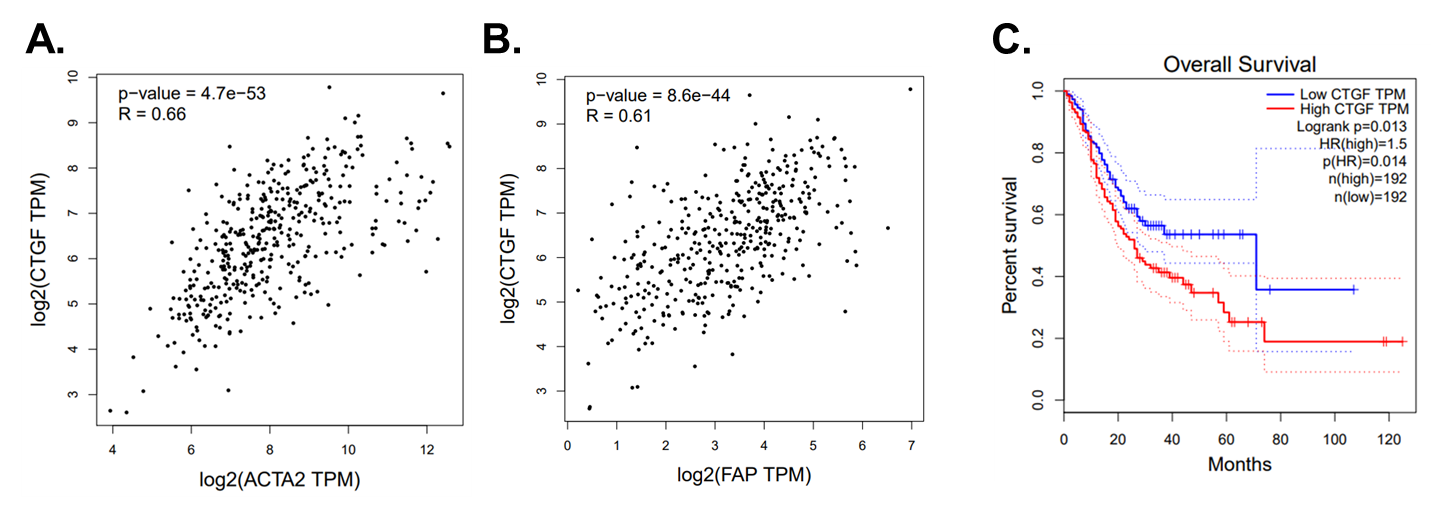


**Supplementary figure 2. CTGF expression is correlated with the expression of CAFs marker genes and its high expression is associated with poor prognosis of GC patients.**

**A and B.** The correlation between the expression of CTGF and CAFs marker genes, ACTA2 (A) and FAP (B), in the TCGA database for stomach adenocarcinoma (STAD), was assessed using the Gene Expression Profiling Interactive Analysis (GEPIA) online tool (<http://gepia.cancer-pku.cn/>). **C.** Kaplan-Meier survival curves were generated to compare the overall survival (OS) of STAD patients with high and low CTGF expression using GEPIA.


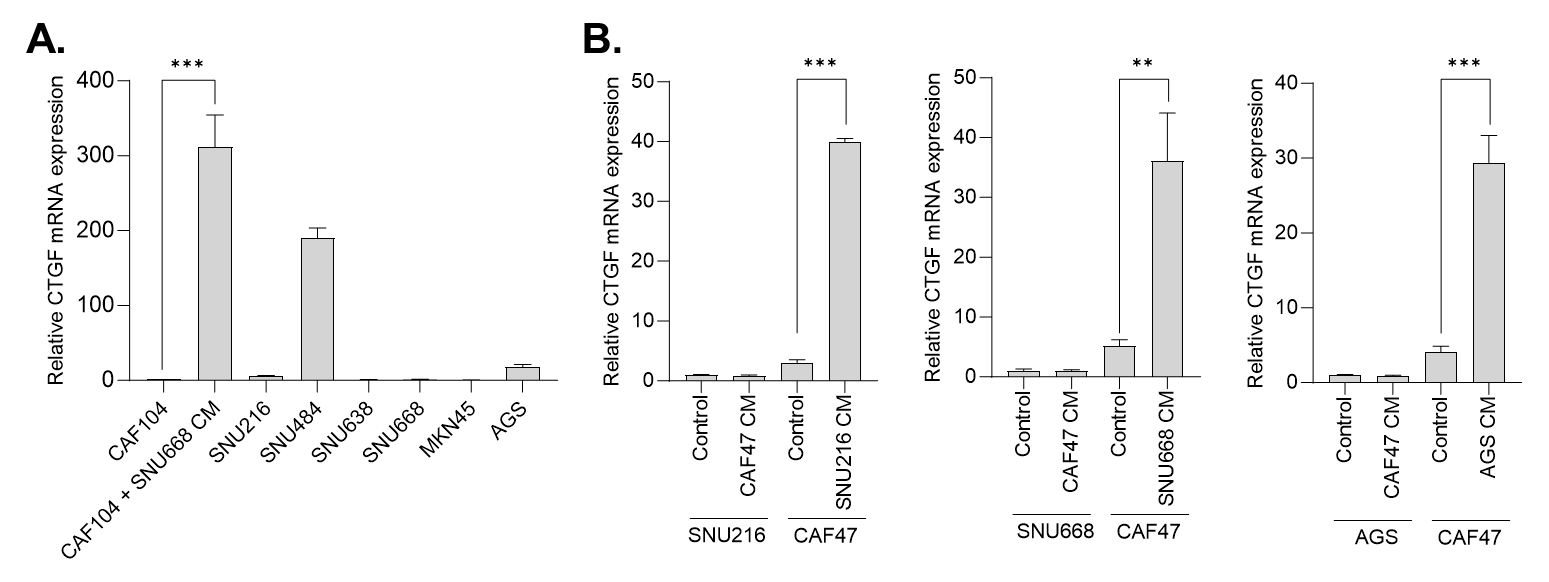


**Supplementary figure 3. CTGF expression in GC cell lines.**

The mRNA expression of CTGF was evaluated in untreated GC cells (A) and GC cells stimulated with CAF CM for 6 hours (B) using RT-qPCR. CAF samples stimulated by GC CM were used as positive controls. Error bars indicate standard deviation of representative triplicates from at least three experiments which showed similar results. NS: not significant, *: p < 0.05, **: p < 0.01, ***: p < 0.001.


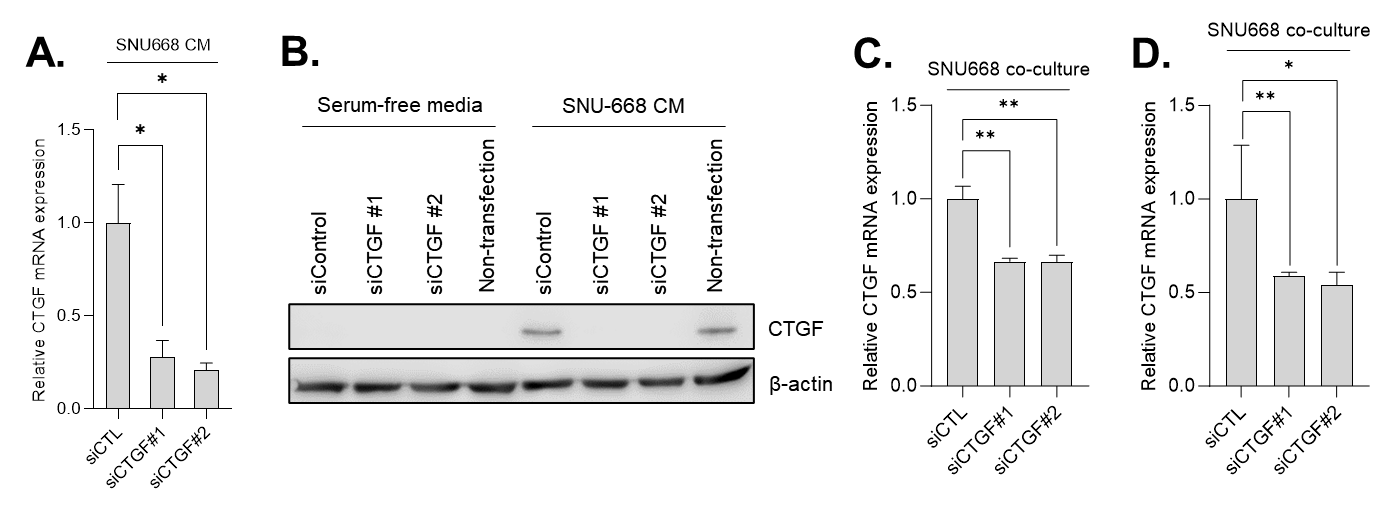


**Supplementary figure 4. The validation of knockdown efficiency of siRNA targeting CTGF.**

CAF47 cells transfected with control or siRNA targeting CTGF were treated with conditioned media (CM) obtained from SNU668 cells for 6 hours and its expression was analyzed by qPCR (A) and western blotting (B). CAF47(C) and CAF104(D) cells transfected with either control siRNA or siRNA targeting CTGF were co-incubated with SNU668 cells for 24 hours, after which their expression was analyzed using qPCR. Error bars indicate standard deviation of representative triplicates from at least three experiments which showed similar results. NS: not significant, *: p < 0.05, **: p < 0.01, ***: p < 0.001.

**Supplementary figure 5. The effect of CTGF knockdown on the viability of SNU484 cells.**

SNU484 cells transfected with control or siRNA targeting CTGF were cultured for 72 hours and then cell viability was analyzed by MTT assay.


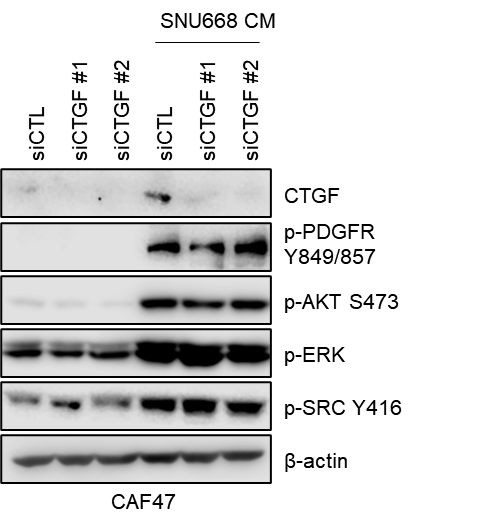


**Supplementary figure 6. CTGF knockdown fails to inhibit GC-stimulated signaling pathways in CAFs.**

CAF47 cells transfected with control siRNA or CTGF targeting siRNA were treated with SNU668 CM for 6 hours. The expressions of CTGF (35 KDa), PDGFR (190 KDa), AKT (60 KDa), ERK (42/44 KDa) and SRC (60 KDa) were analyzed by western blotting.

**Supplementary figure 7. CTGF knockdown fails to inhibit GC-stimulated proliferation of CAFs.**

CAF47 cells transfected with control or siRNA targeting CTGF were treated with conditioned media (CM) obtained from SNU668 cells for 72 hours and then cell viability was analyzed by MTT assay. Error bars indicate standard deviation of representative triplicates from at least three experiments which showed similar results. NS: not significant, *: p < 0.05, **: p < 0.01, ***: p < 0.001.

**Supplementary figure 8. Recombinant CTGF fails to promote proliferation of GC cells.**

SNU668 cells were incubated with different concentrations of recombinant human (rh) CTGF for 72 hours and then cell viability was analyzed by MTT assay.


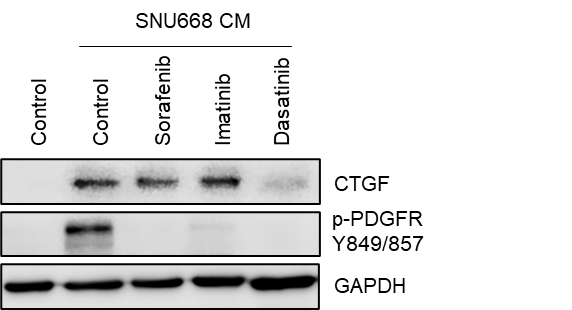


**Supplementary figure 9.** **The effect of PDGFR inhibitors on GC-induced CTGF expression in CAFs.**

CAF47 cells were treated with SNU668 CM along with 1 μM of sorafenib, imatinib and dasatinib for 6 hours, and the expression of CTGF (35 KDa), p-PDGFR (190 KDa) was analyzed by western blotting.

**Supplementary figure 10.** **The effect of dasatinib and TKIs which have been utilized in clinical studies for GC treatment on CTGF expression in activated CAFs.**

CAF47 cells were treated with SNU668 CM along with 0.1 μM of indicated TKIs for 6 hours, and CTGF expression was analyzed using qPCR.

**Supplementary figure 11. Dasatinib inhibits CTGF expression in activated CAFs.**

CAF47 cells were treated with SNU668 CM and varying doses of dasatinib (10 to 1000 nM) for 6 hours. The mRNA expressions of CTGF were analyzed using RT-qPCR. Error bars indicate standard deviation of representative triplicates from at least three experiments which showed similar results. NS: not significant, *: p < 0.05, **: p < 0.01, ***: p < 0.001.


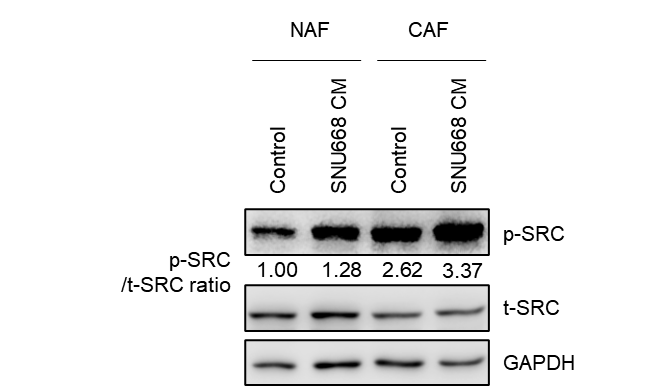


**Supplementary figure 12. Differential SRC activation in GC-stimulated NAFs and CAFs.**

NAF or CAF cells were stimulated with SNU668 CM for 6 hours, then tyrosine phosphorylated and total SRC (60 KDa) levels were analyzed by western blotting.

**Supplementary figure 13.** **Dasatinib and saracatinib decrease GC-induced CTGF expression in CAF104.**

CAF104 cells were treated with SNU668 CM along with 500 nM of dasatinib or saracatinib for 6 hours, and the expression of CTGF was analyzed by RT-qPCR. Error bars indicate standard deviation of representative triplicates from at least three experiments which showed similar results. NS: not significant, *: p < 0.05, **: p < 0.01, ***: p < 0.001.


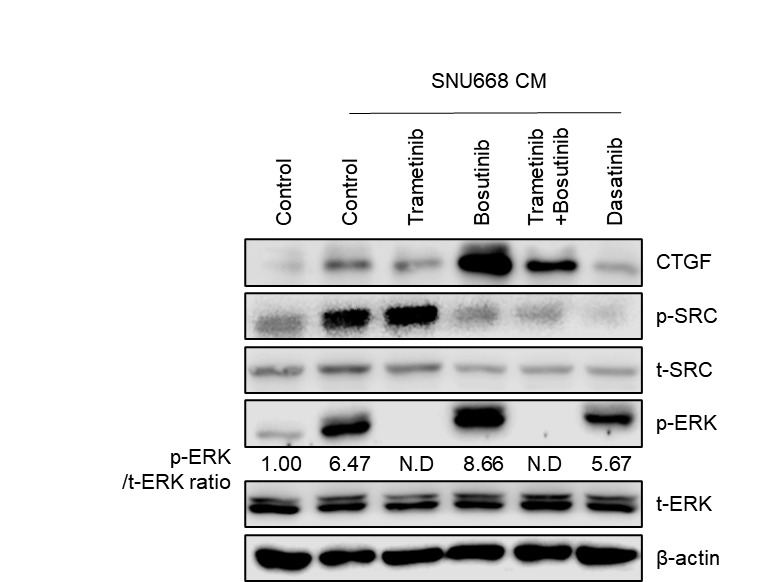


**Supplementary figure 14. Combination of trametinib and bosutinib decreases GC-induced CTGF expression in CAF47.**

CAFs were treated with SNU668 CM along with 1 μM of MEK inhibitor (trametinib), 1 μM of SRC inhibitors (bosutinib or dasatinib), or trametinib : bosutinib combination for 6 hours, then the expression of CTGF and phospho/ total SRC (60 KDa) and ERK (42/44 KDa) was analyzed by western blotting.


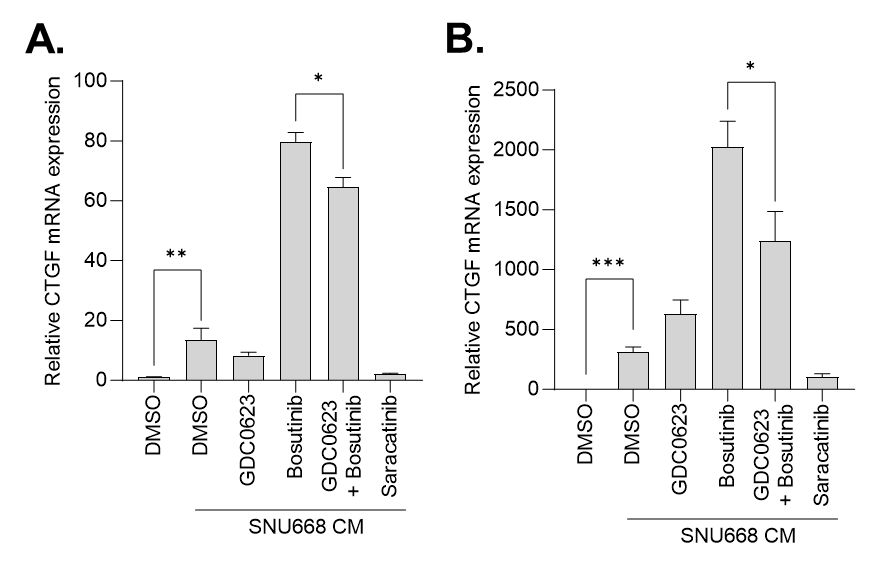
**Supplementary figure 15. Combination of GDC0623 and bosutinib decreases GC-induced CTGF expression in CAFs.**

CAF47 (A) and CAF104 (B) cells were treated with SNU668 CM along with 1 μM of MEK inhibitor (GDC0623), 1 μM of SRC inhibitors (bosutinib or saracatinib), or GDC0623 : bosutinib combination for 6 hours, the expression of CTGF was analyzed by qPCR. Error bars indicate standard deviation of representative triplicates from at least three experiments which showed similar results. NS: not significant, *: p < 0.05, **: p < 0.01, ***: p < 0.001.


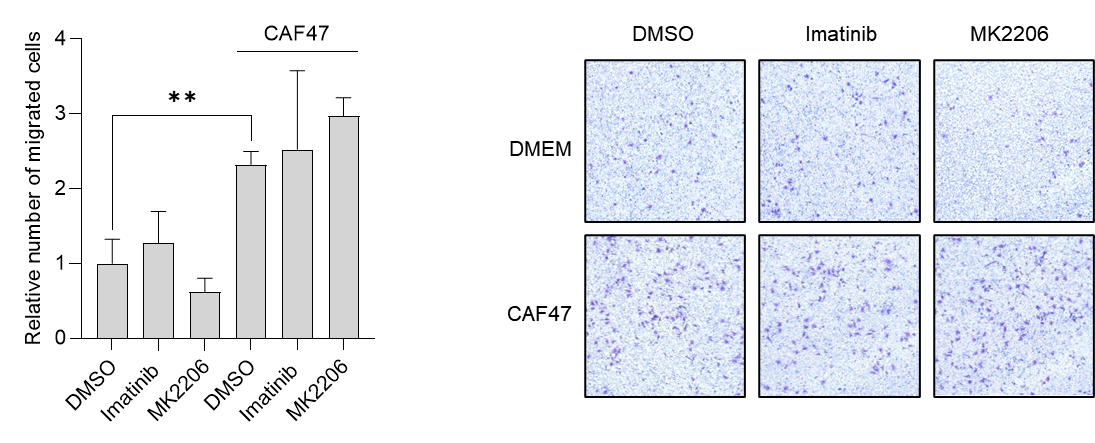
**Supplementary figure 16. Imatinib and MK2206 fail to inhibit GC cell migration promoted by activated CAFs.** The migration of SNU668 cells co-cultured with imatinib (5 μM, PDGFR inhibitor) or MK2206 (1 μM, AKT inhibitor)-treated CAF47 cells was analyzed by transwell migration assay. Representative photographs of migrated cells on the membrane (magnification, 200x) are shown. The error bars indicate the standard deviation of representative triplicates from at least three experiments which showed similar results. *: p < 0.05, **: p < 0.01, ***: p < 0.001.


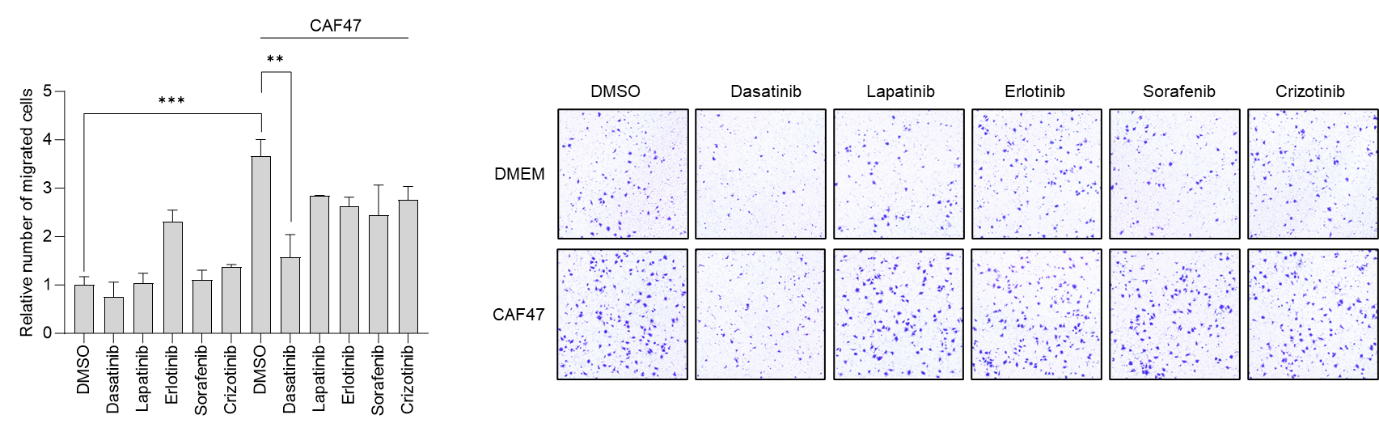


**Supplementary figure 17. The effect of dasatinib and TKIs** **which have been utilized in clinical studies for GC treatment on CAFs-induced GC cell migration.** The migration of SNU668 cells co-cultured with 0.1 μM of indicated TKIs was analyzed by transwell migration assay. Representative photographs of migrated cells on the membrane (magnification, 200x) are shown. The error bars indicate the standard deviation of representative triplicates from at least three experiments which showed similar results. *: p < 0.05, **: p < 0.01, ***: p < 0.001.

.**
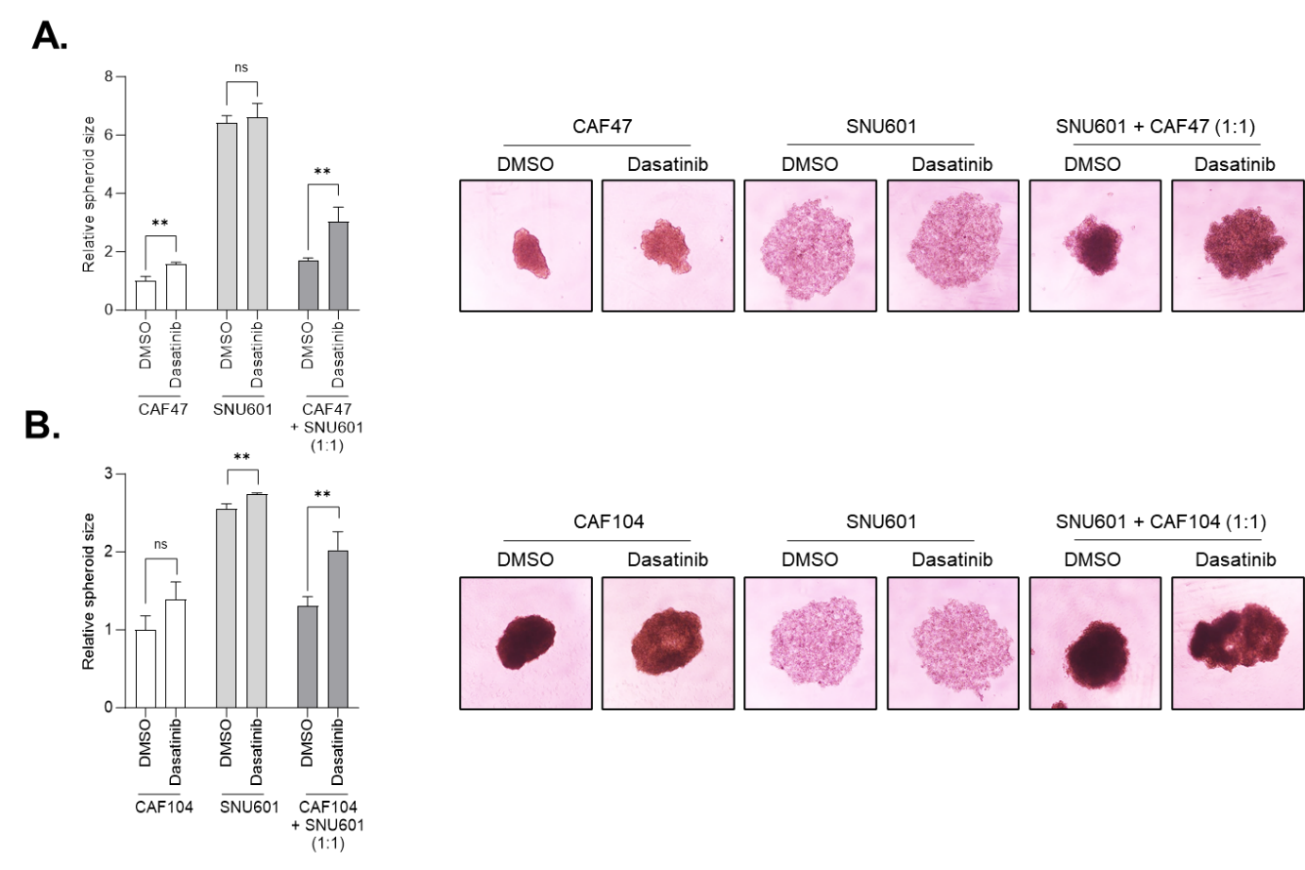
**

**Supplementary figure 18. Dasatinib inhibits tumor aggregate formation of GC cells promoted by activated CAFs.** Mono- or bicellular 3D spheroids were generated using SNU601 cells with CAF47 (A) or CAF104 (B) cells. Dasatinib was treated at a concentration of 1 µM. Error bars indicate the standard deviation of representative triplicates from at least three experiments, which showed similar results. NS: not significant, *: p < 0.05, **: p < 0.01, ***: p < 0.001.


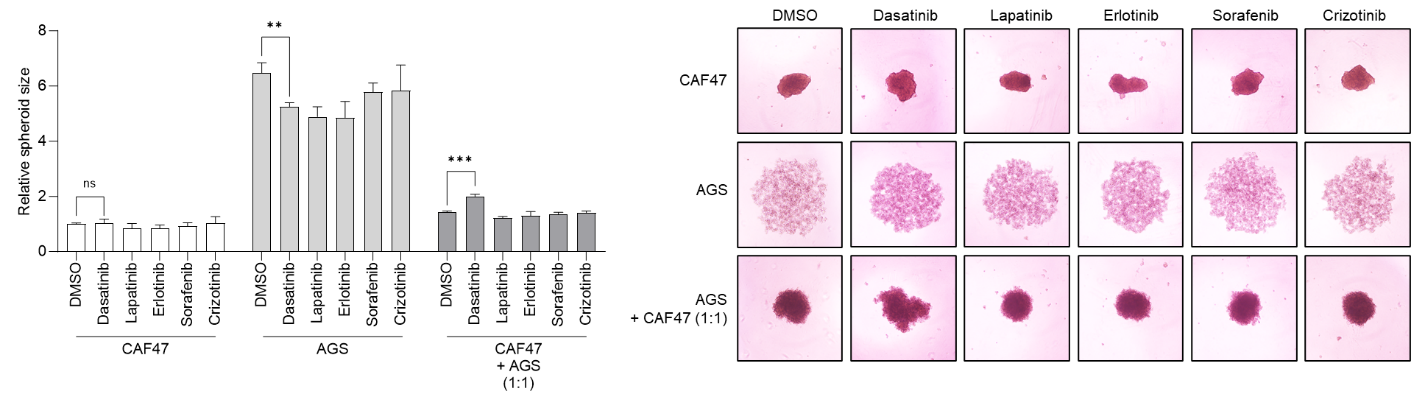


**Supplementary figure 19. The effect of dasatinib and TKIs which have been utilized in clinical studies for GC treatment on CAFs-induced tumor spheroid compactness.** Mono- or bicellular 3D spheroids were generated using AGS cells with CAF47 cells. TKIs were treated at a concentration of 0.1 µM. Error bars indicate the standard deviation of representative triplicates from at least three experiments, which showed similar results. NS: not significant, *: p < 0.05, **: p < 0.01, ***: p < 0.001.

**Supplementary table 1. The sequence of qPCR primers used for this study.**

| Gene | Primer sequence |
| --- | --- |
| ACTA2 (F) | CAG ATG TGG ATC AGC AAA CA |
| ACTA2 (R) | TGG CTA GGA ATG ATT TGG AA |
| CTGF (F) | GTC CAG ACC ACA AG TGG AG |
| CTGF (R) | AGT ACG GAT GCA CTT TTT GC |
| CXCL1 (F) | ACT CAA GAA TGG GCG GAA AGC TTG |
| CXCL1 (R) | AGC GAT GCT CAA ACA CAT TAG GCA C |
| MMP1 (F) | ACA CCT CTG ACA TTC ACC AAG |
| MMP1 (R) | ATG AGC CGC AAC ACG ATG |
| TGFβ2 (F) | TCG ACA GCA AAG TTG TGA AA |
| TGFβ2 (R) | GCC ATC AAT ACC TGC AAA TC |
| GAPDH (F) | AA GGG TCA TCA TCT CTG CCC |
| GAPDH (R) | GT GAT GGC ATG GAC TGT GGT |
