# Supplementary material for: Characterization of gastric cancer-stimulated signaling pathways and function of CTGF in cancer-associated fibroblasts
Source: Cell Commun Signal. 2024 Jan 2;22:8. doi: 10.1186/s12964-023-01396-7 (PMC10763493; doi:10.1186/s12964-023-01396-7)

Figure 1A.

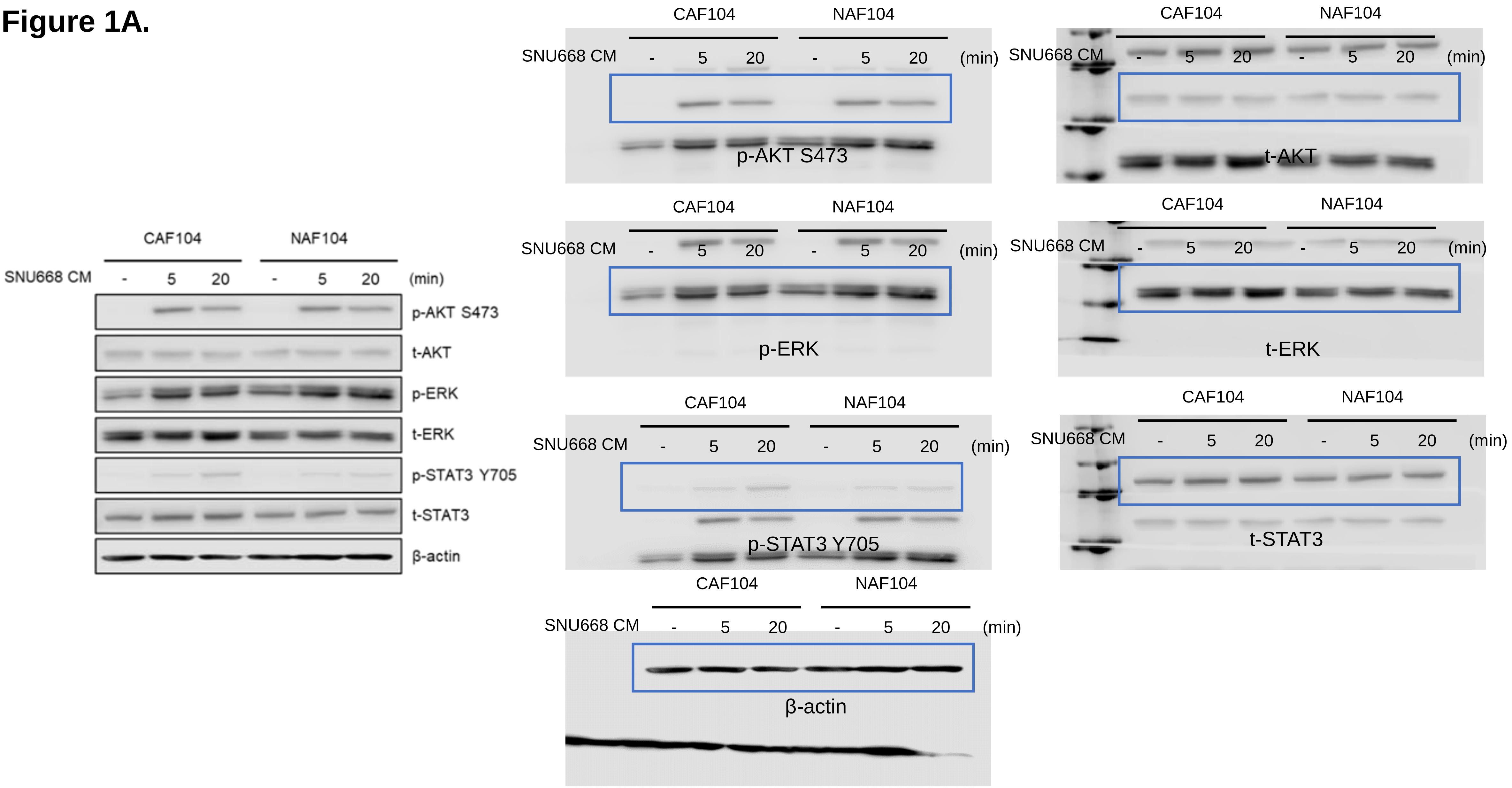
CAF104
NAF104
SNU668 CM
- 5 20 - 5 20 (min)
p-AKT S473
t-AKT
p-ERK
t-ERK
p-STAT3 Y705
t-STAT3
β-actin

Figure 1B.

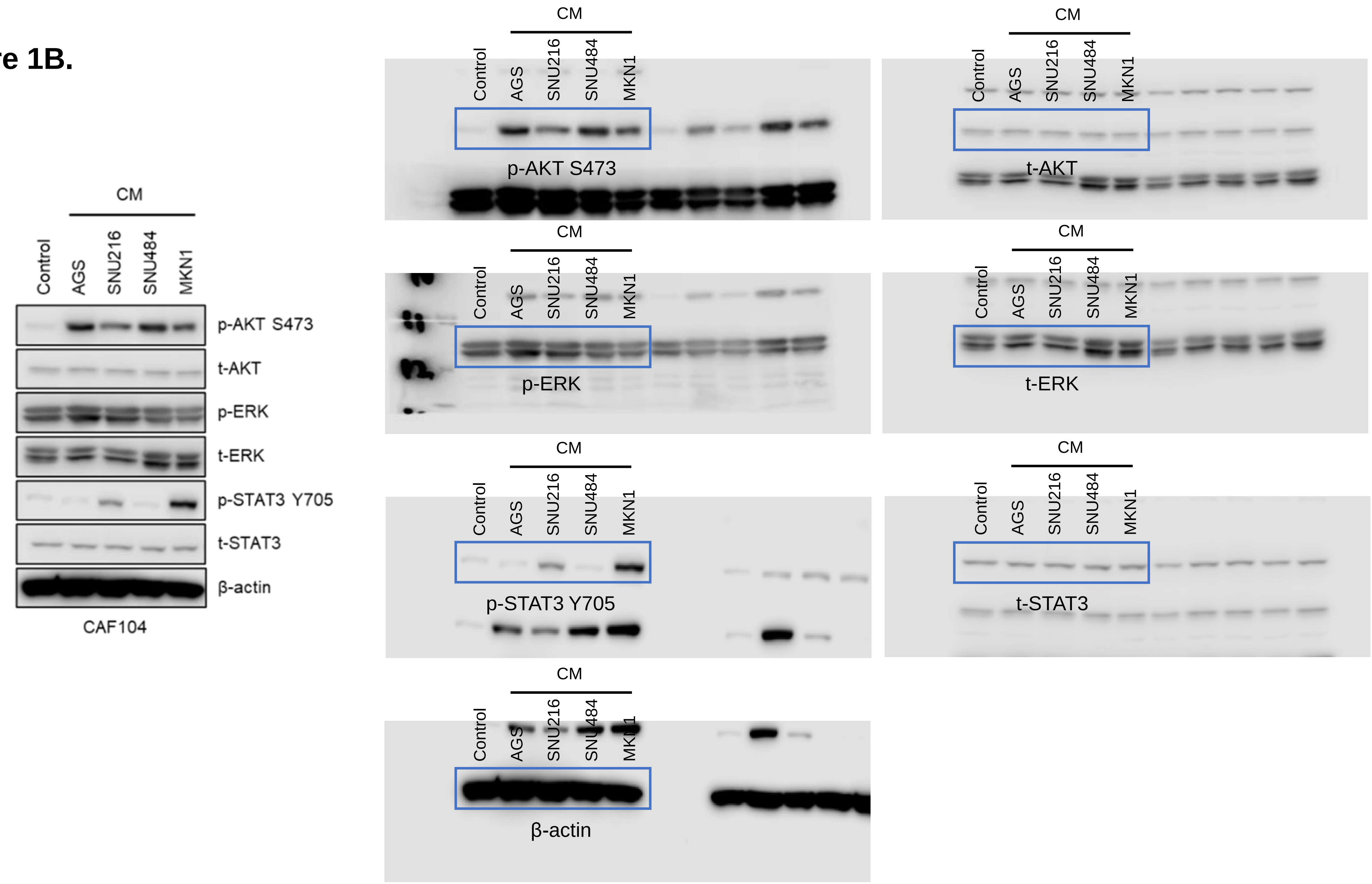
CM
Control
AGS
SNU216
SNU484
MKN1
p-AKT S473
t-AKT
p-ERK
t-ERK
p-STAT3 Y705
t-STAT3
β-actin
CAF104
p-AKT S473
t-AKT
p-ERK
t-ERK
p-STAT3 Y705
t-STAT3
β-actin

Figure 1C.

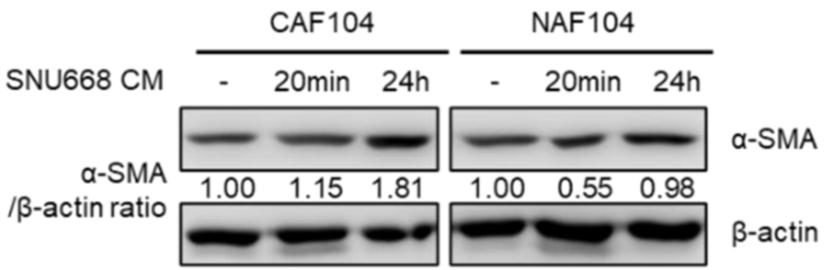
CAF104
NAF104
SNU668 CM
-
20min
24h
-
20min
24h
α-SMA
α-SMA /β-actin ratio
1.00
1.15
1.81
1.00
0.55
0.98
β-actin

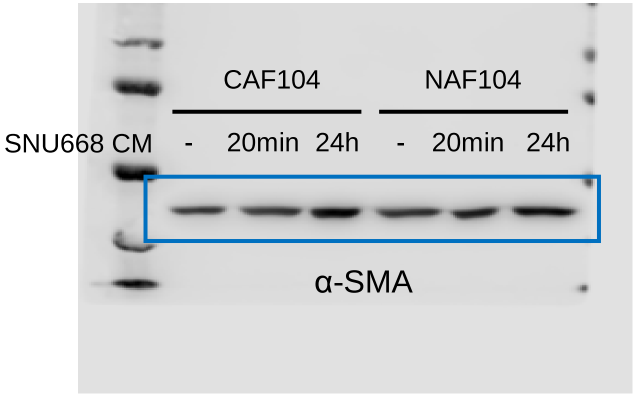
CAF104
NAF104
SNU668 CM
-
20min
24h
-
20min
24h
α-SMA

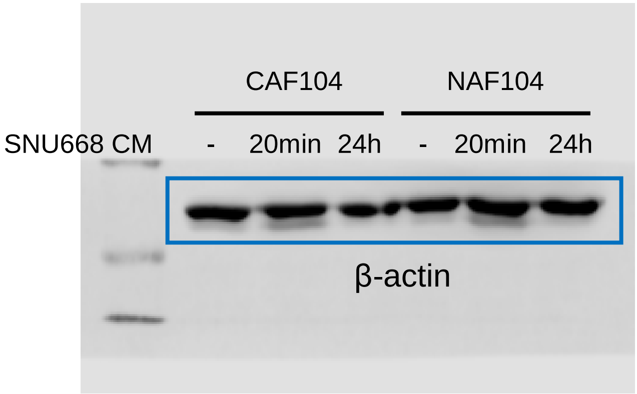
CAF104
NAF104
SNU668 CM
-
20min
24h
-
20min
24h
β-actin

Figure 1D.

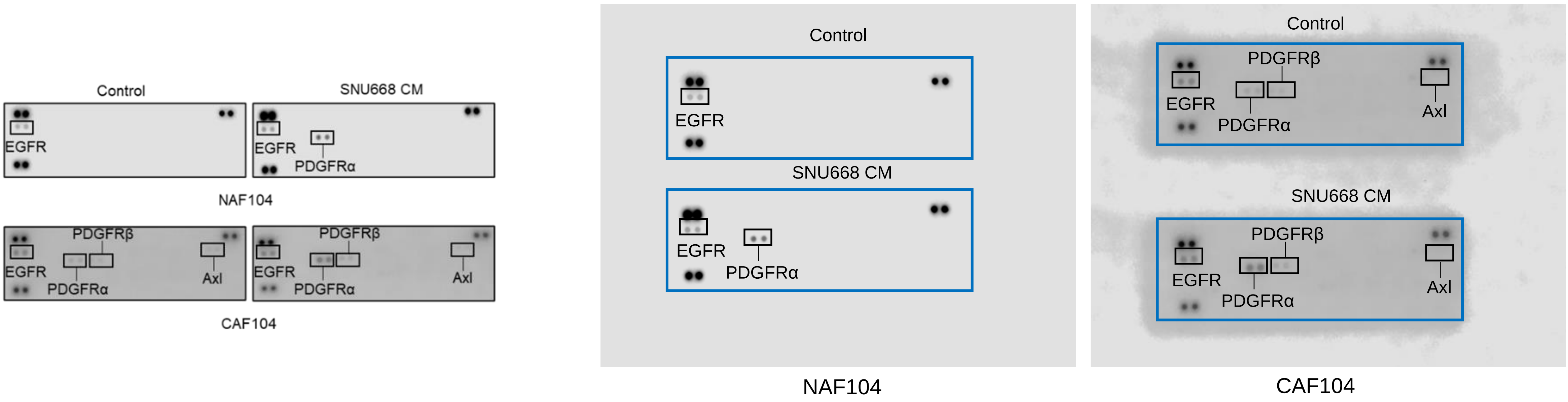

Figure 1E.

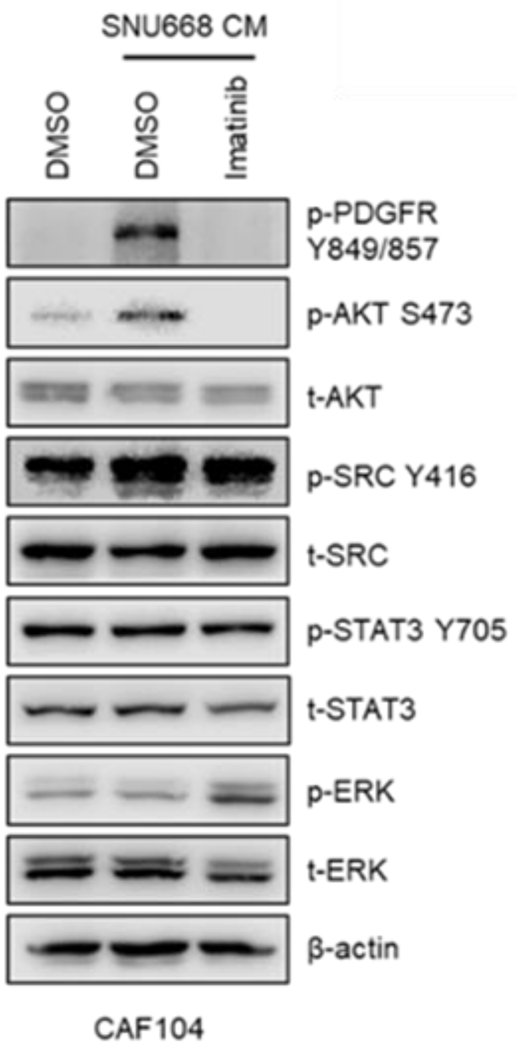

SNU668 CM
DMSO
DMSO
Imatinib
p-PDGFR Y849/857
p-AKT S473
t-AKT
p-SRC Y416
t-SRC
p-STAT3 Y705
t-STAT3
p-ERK
t-ERK
β-actin
CAF104

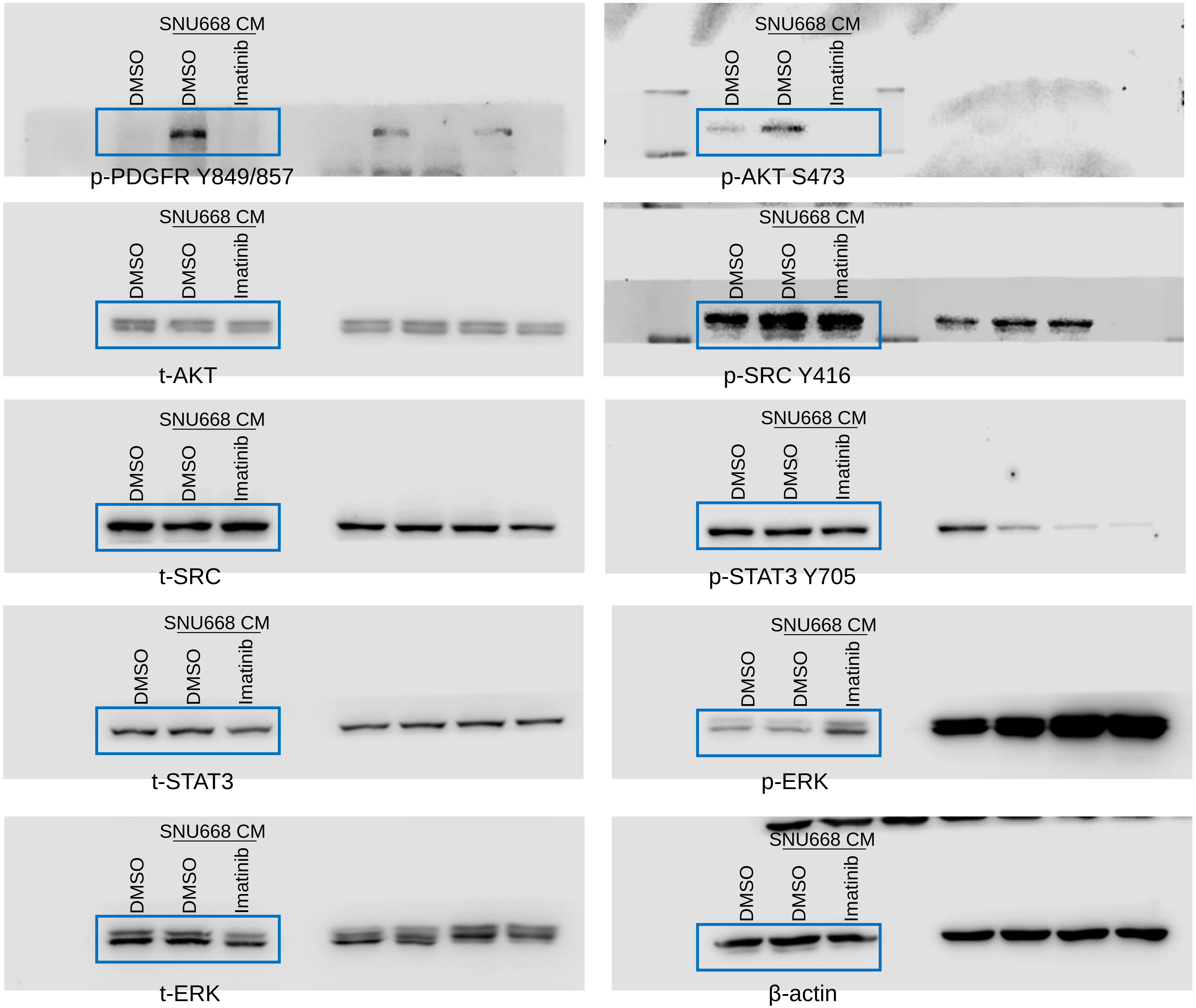

SNU668 CM
DMSO
DMSO
Imatinib
p-PDGFR Y849/857
p-AKT S473
t-AKT
p-SRC Y416
t-SRC
p-STAT3 Y705
t-STAT3
p-ERK
t-ERK
β-actin

Figure 1F.

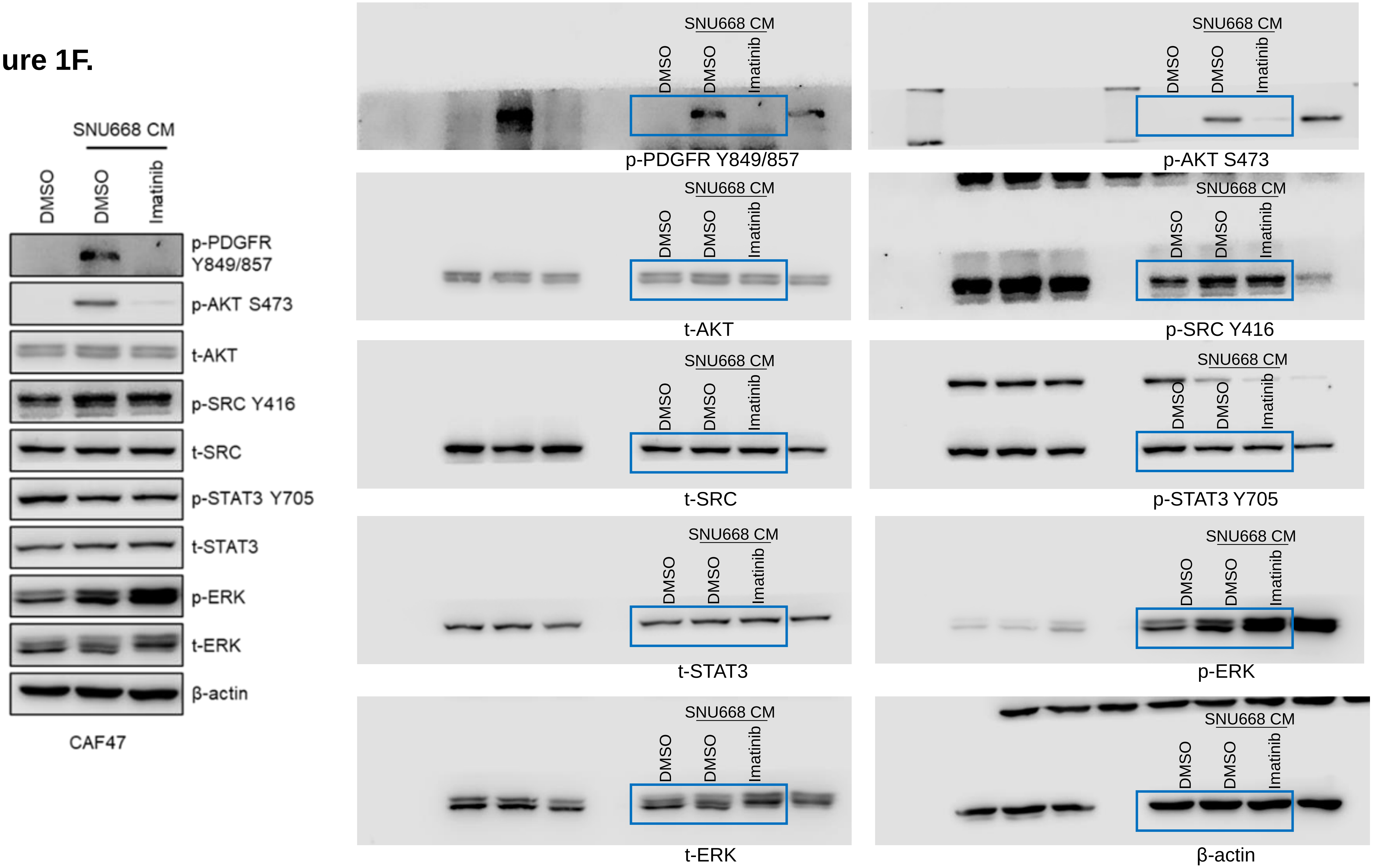
SNU668 CM
DMSO
DMSO
Imatinib
p-PDGFR Y849/857
p-AKT S473
t-AKT
p-SRC Y416
t-SRC
p-STAT3 Y705
t-STAT3
p-ERK
t-ERK
β-actin
CAF47
p-PDGFR Y849/857
p-AKT S473
t-AKT
p-SRC Y416
t-SRC
p-STAT3 Y705
t-STAT3
p-ERK
t-ERK
β-actin

Figure 2D.

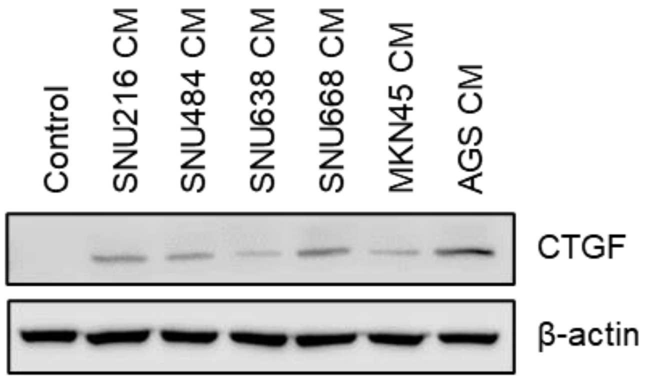

Control
SNU216 CM
SNU484 CM
SNU638 CM
SNU668 CM
MKN45 CM
AGS CM
CTGF
β-actin

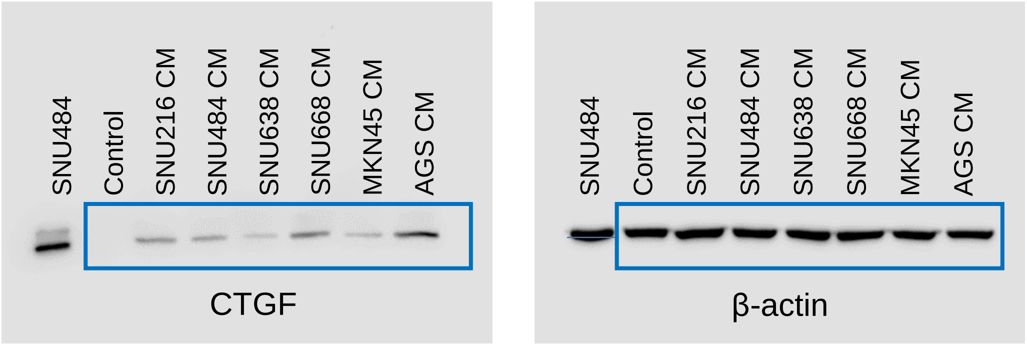

SNU484
Control
SNU216 CM
SNU484 CM
SNU638 CM
SNU668 CM
MKN45 CM
AGS CM
CTGF
SNU484
Control
SNU216 CM
SNU484 CM
SNU638 CM
SNU668 CM
MKN45 CM
AGS CM
β-actin

Figure 2E.

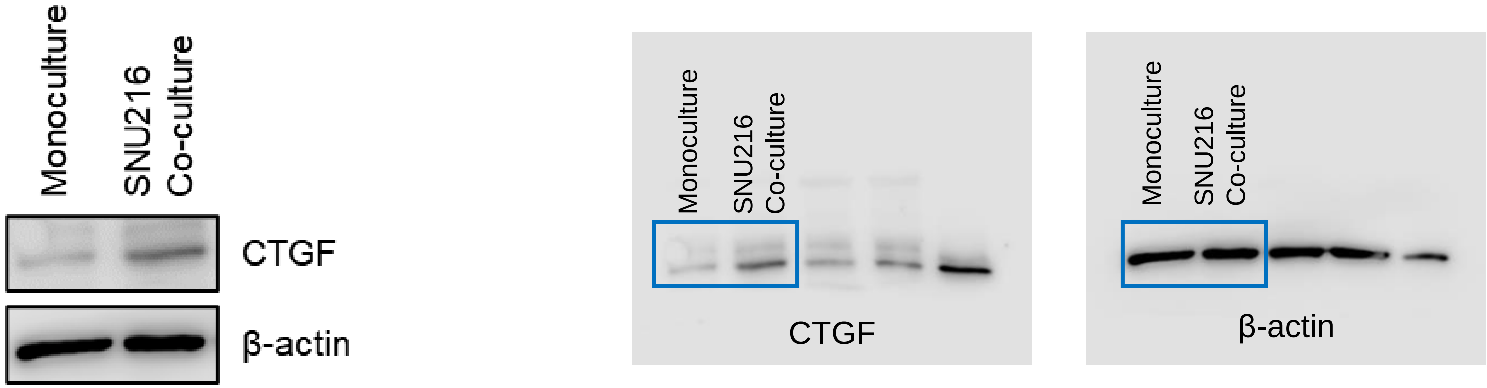
Monoculture
SNU216
Co-culture
CTGF
β-actin
Monoculture
SNU216
Co-culture
CTGF
Monoculture
SNU216
Co-culture
β-actin

Figure 2F.

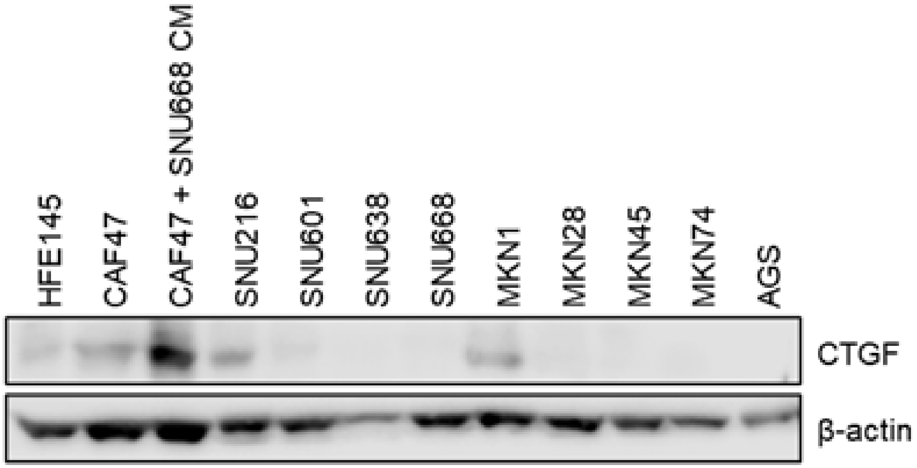
HFE145
CAF47
CAF47 + SNU668 CM
SNU216
SNU601
SNU638
SNU668
MKN1
MKN28
MKN45
MKN74
AGS
CTGF
β-actin

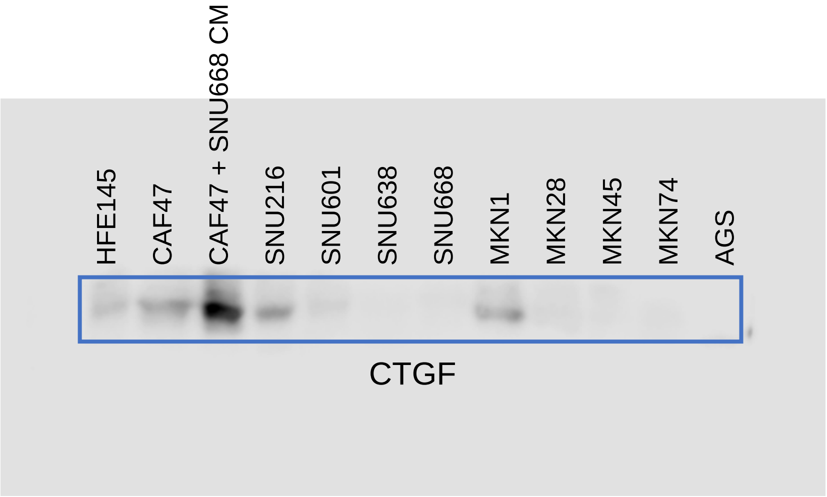
HFE145
CAF47
CAF47 + SNU668 CM
SNU216
SNU601
SNU638
SNU668
MKN1
MKN28
MKN45
MKN74
AGS
CTGF

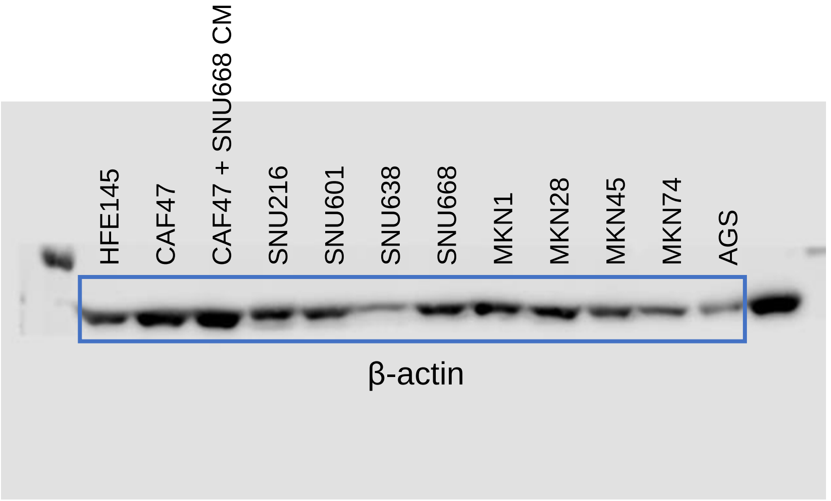
HFE145
CAF47
CAF47 + SNU668 CM
SNU216
SNU601
SNU638
SNU668
MKN1
MKN28
MKN45
MKN74
AGS
β-actin

Figure 2G to 2I

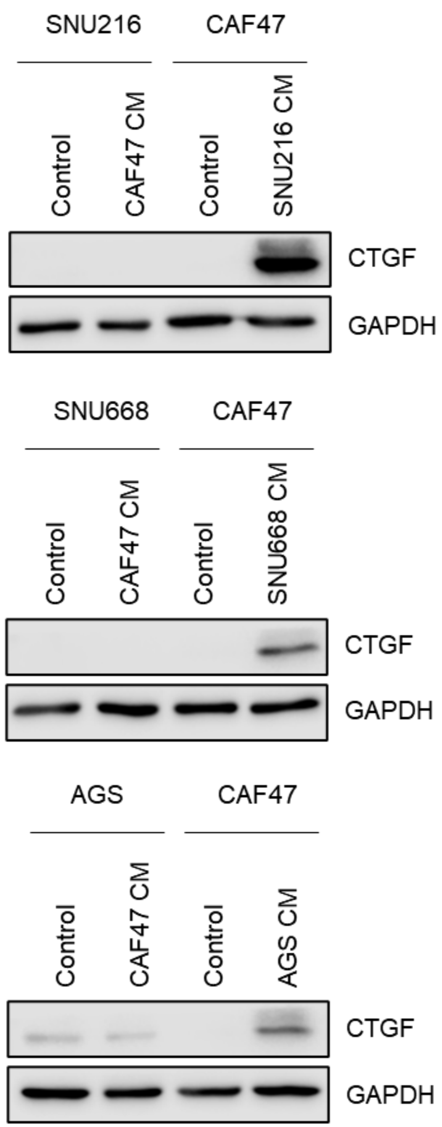

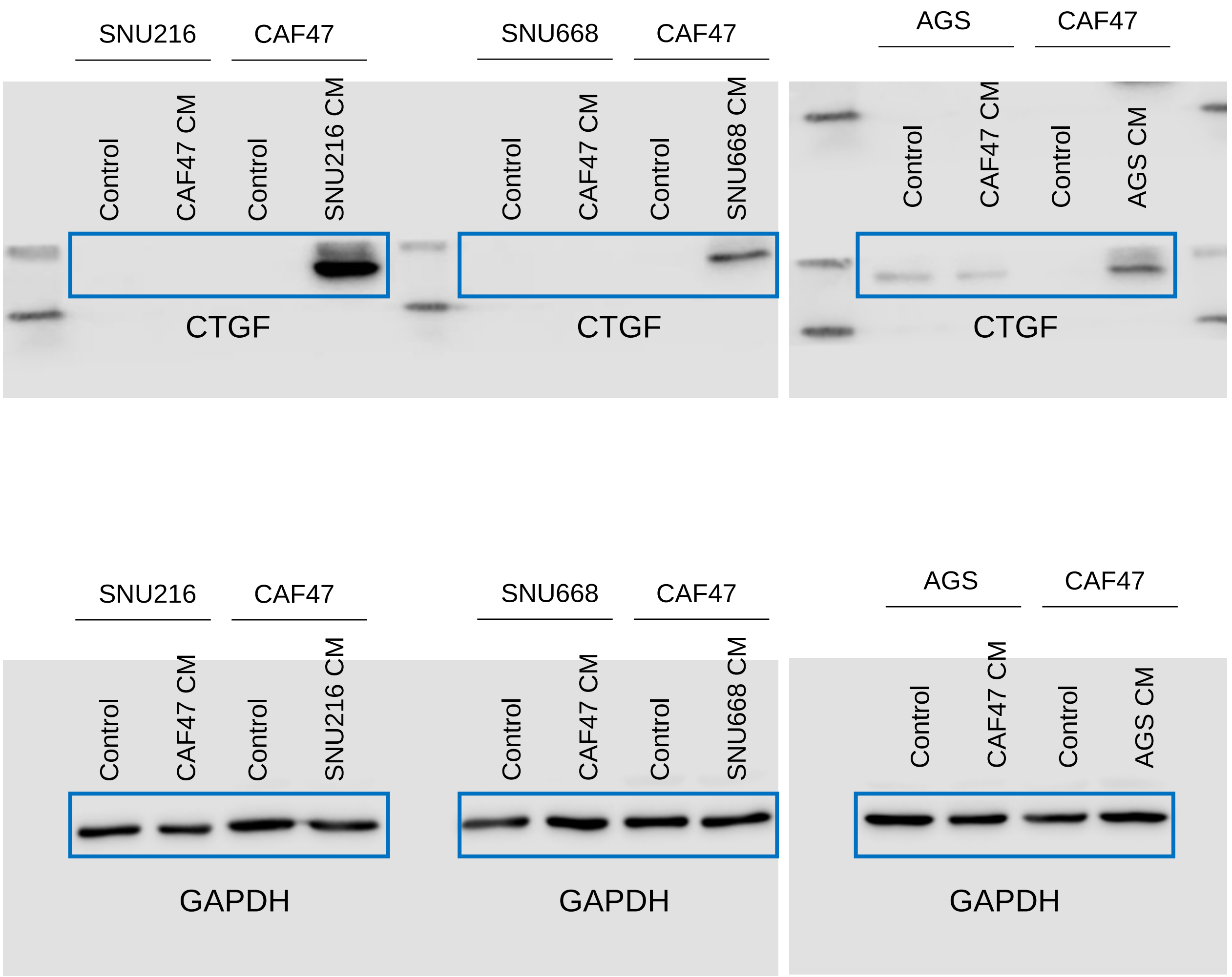

Figure 4A.

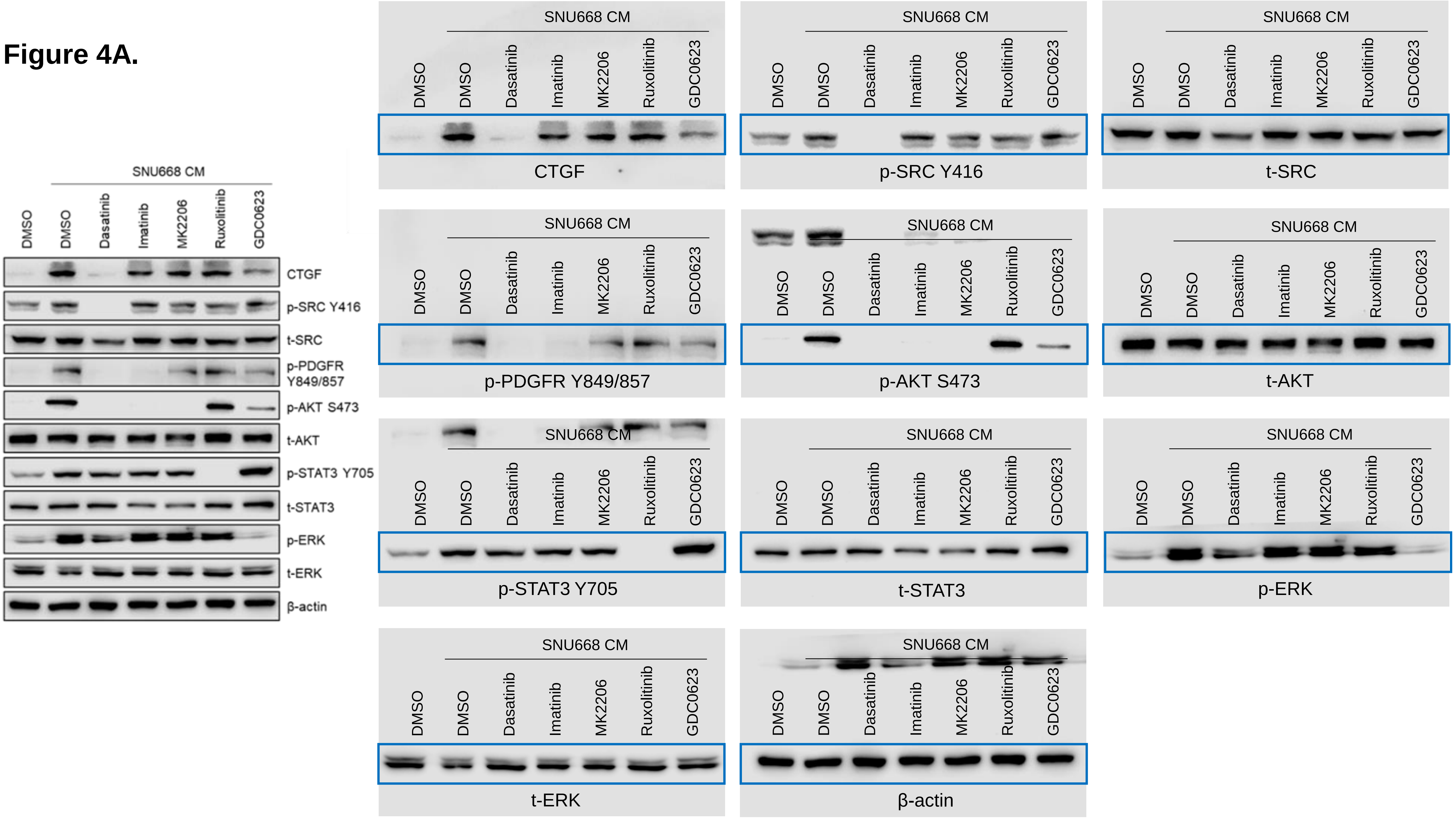
SNU668 CM
DMSO
DMSO
Dasatinib
Imatinib
MK2206
Ruxolitinib
GDC0623
CTGF
p-SRC Y416
t-SRC
p-PDGFR Y849/857
p-AKT S473
t-AKT
p-STAT3 Y705
t-STAT3
p-ERK
t-ERK
β-actin

Figure 4C.

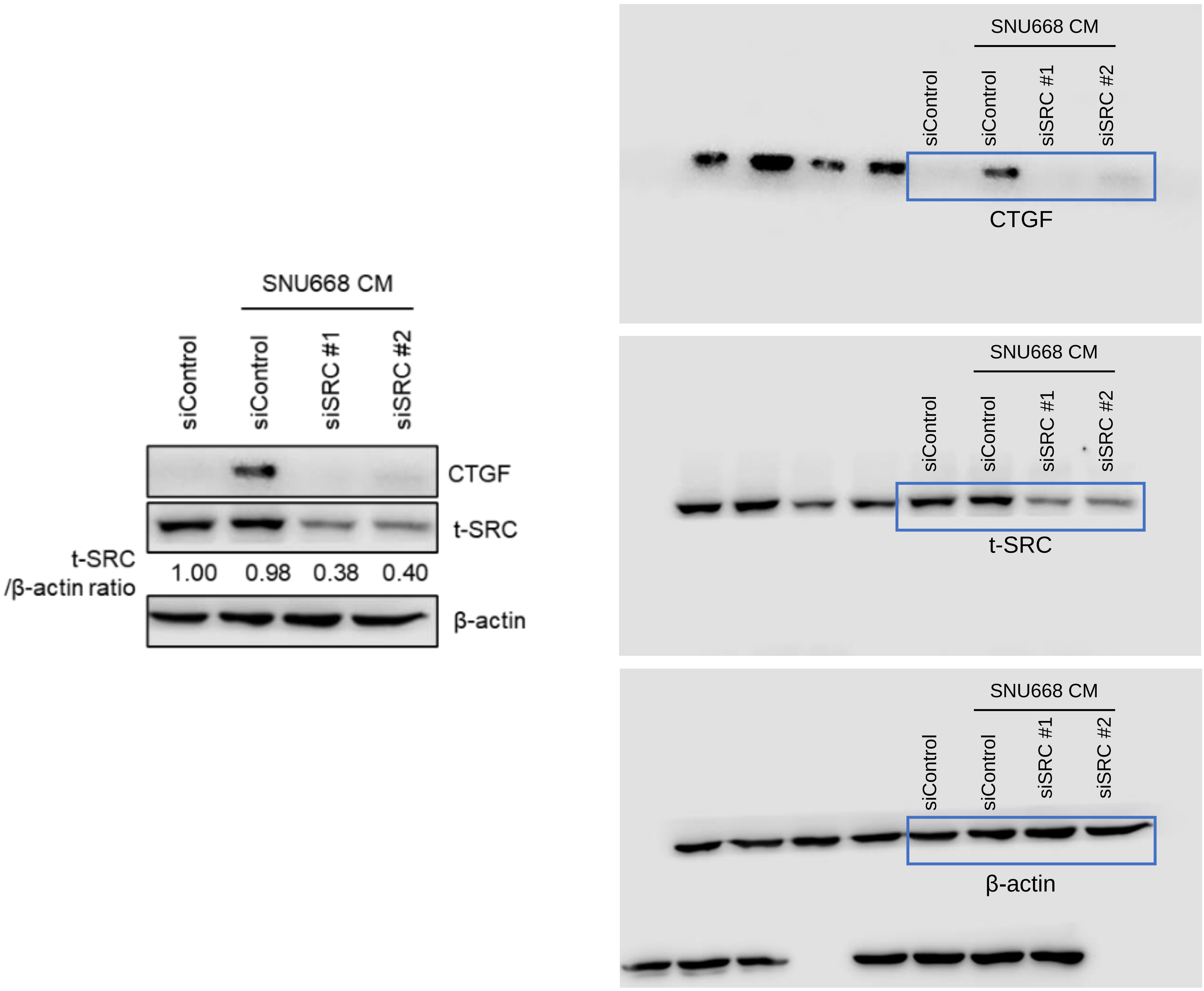

SNU668 CM
siControl
siControl
siSRC #1
siSRC #2
CTGF
t-SRC
t-SRC
/β-actin ratio
1.00
0.98
0.38
0.40
β-actin
SNU668 CM
siControl
siControl
siSRC #1
siSRC #2
CTGF
SNU668 CM
siControl
siControl
siSRC #1
siSRC #2
t-SRC
SNU668 CM
siControl
siControl
siSRC #1
siSRC #2
β-actin

Figure 4E.

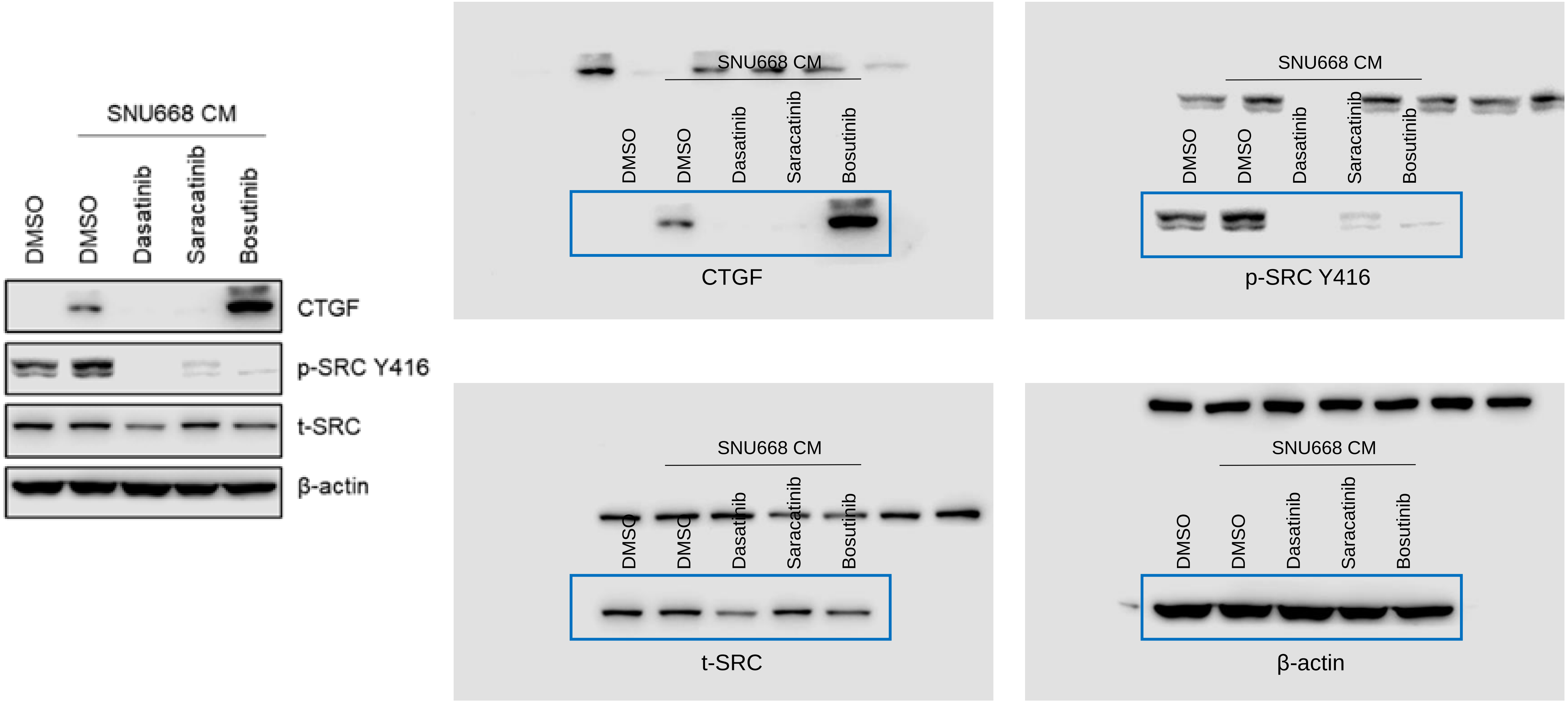
SNU668 CM
DMSO
DMSO
Dasatinib
Saracatinib
Bosutinib
CTGF
p-SRC Y416
t-SRC
β-actin
SNU668 CM
DMSO
DMSO
Dasatinib
Saracatinib
Bosutinib
CTGF
SNU668 CM
DMSO
DMSO
Dasatinib
Saracatinib
Bosutinib
p-SRC Y416
SNU668 CM
DMSO
DMSO
Dasatinib
Saracatinib
Bosutinib
t-SRC
SNU668 CM
DMSO
DMSO
Dasatinib
Saracatinib
Bosutinib
β-actin

Figure 5B.

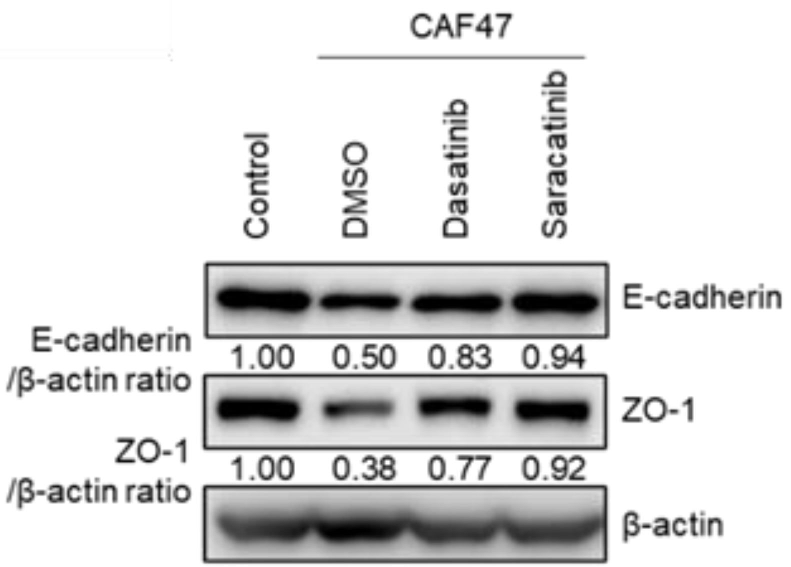

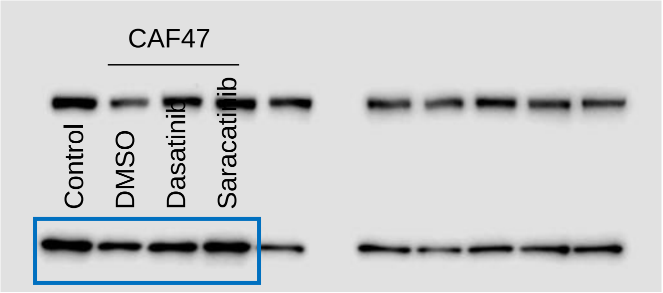

E-cadherin

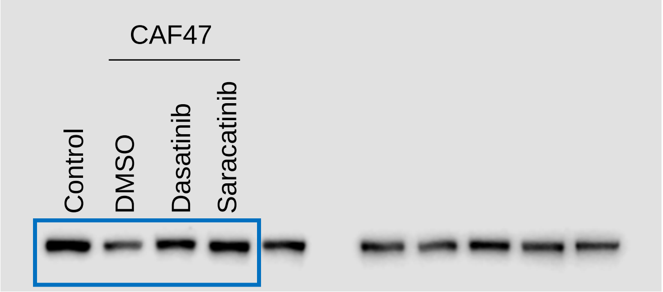

ZO-1

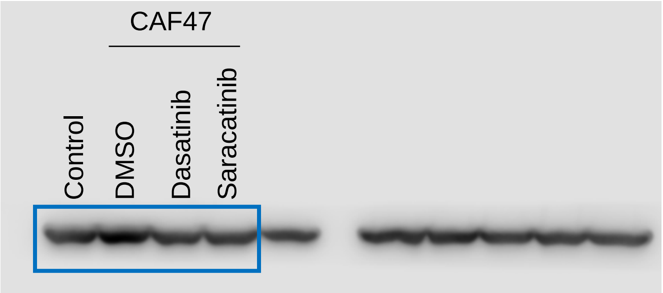

β-actin

Supplementary figure 1.

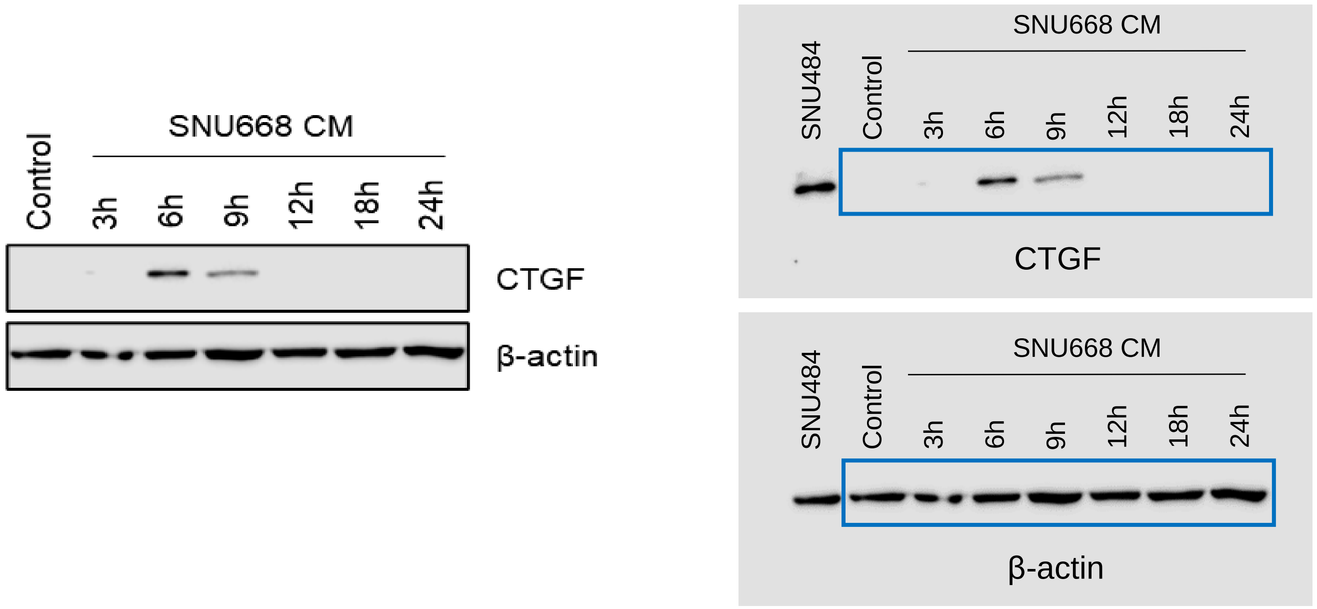

Supplementary figure 4B.

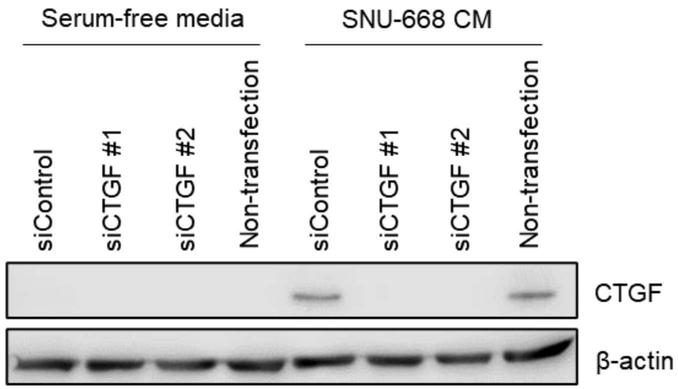

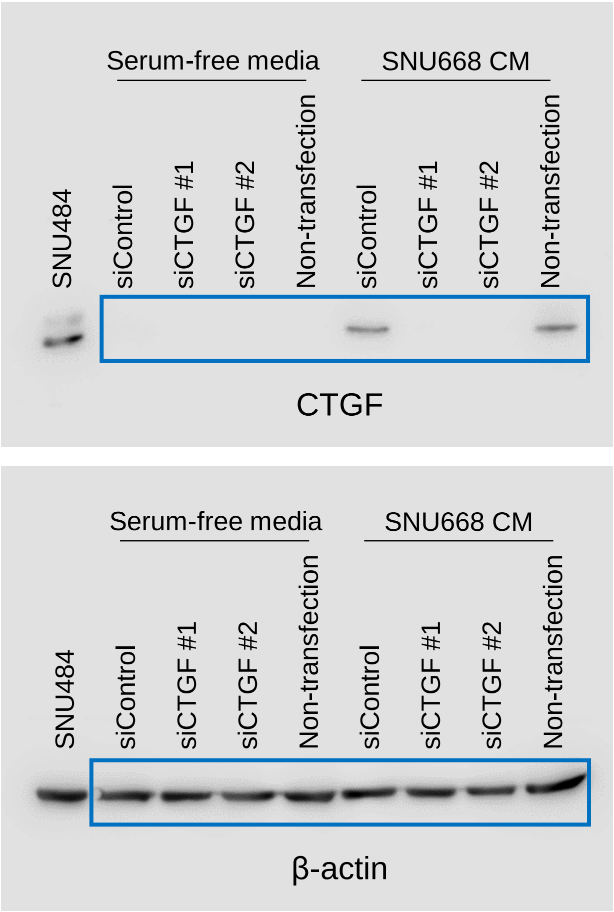

Supplementary figure 6.

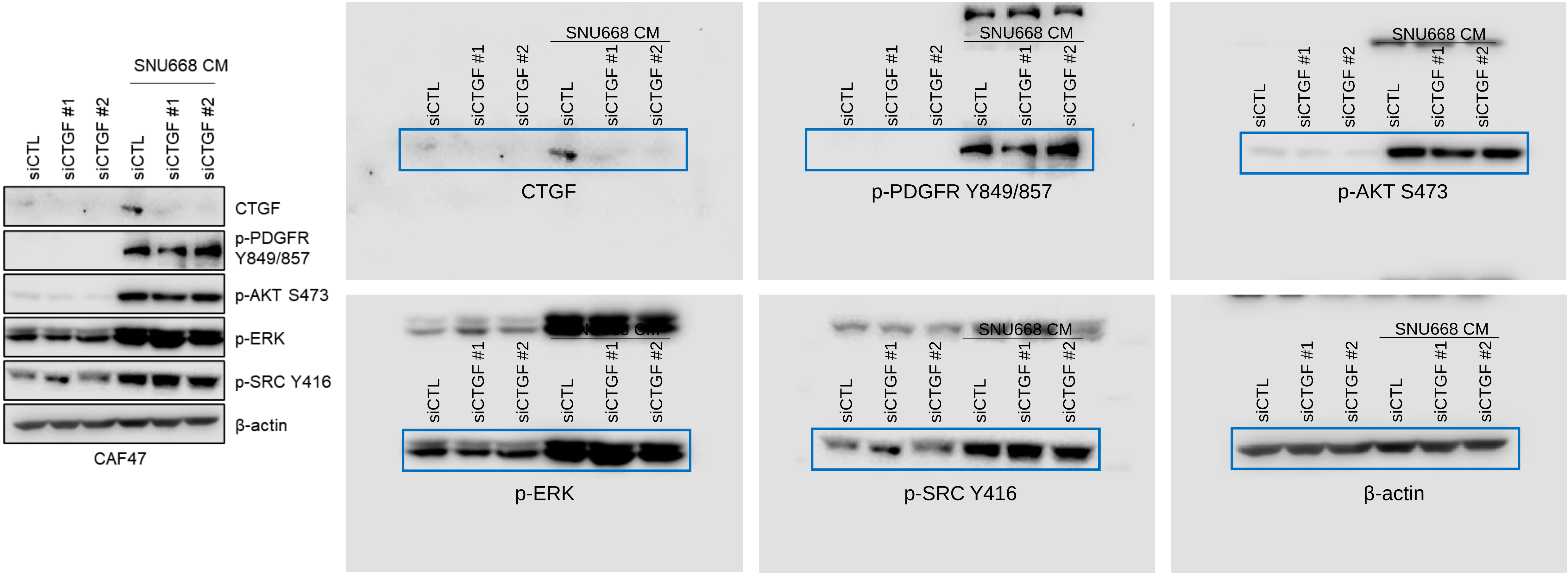

Supplementary figure 9.

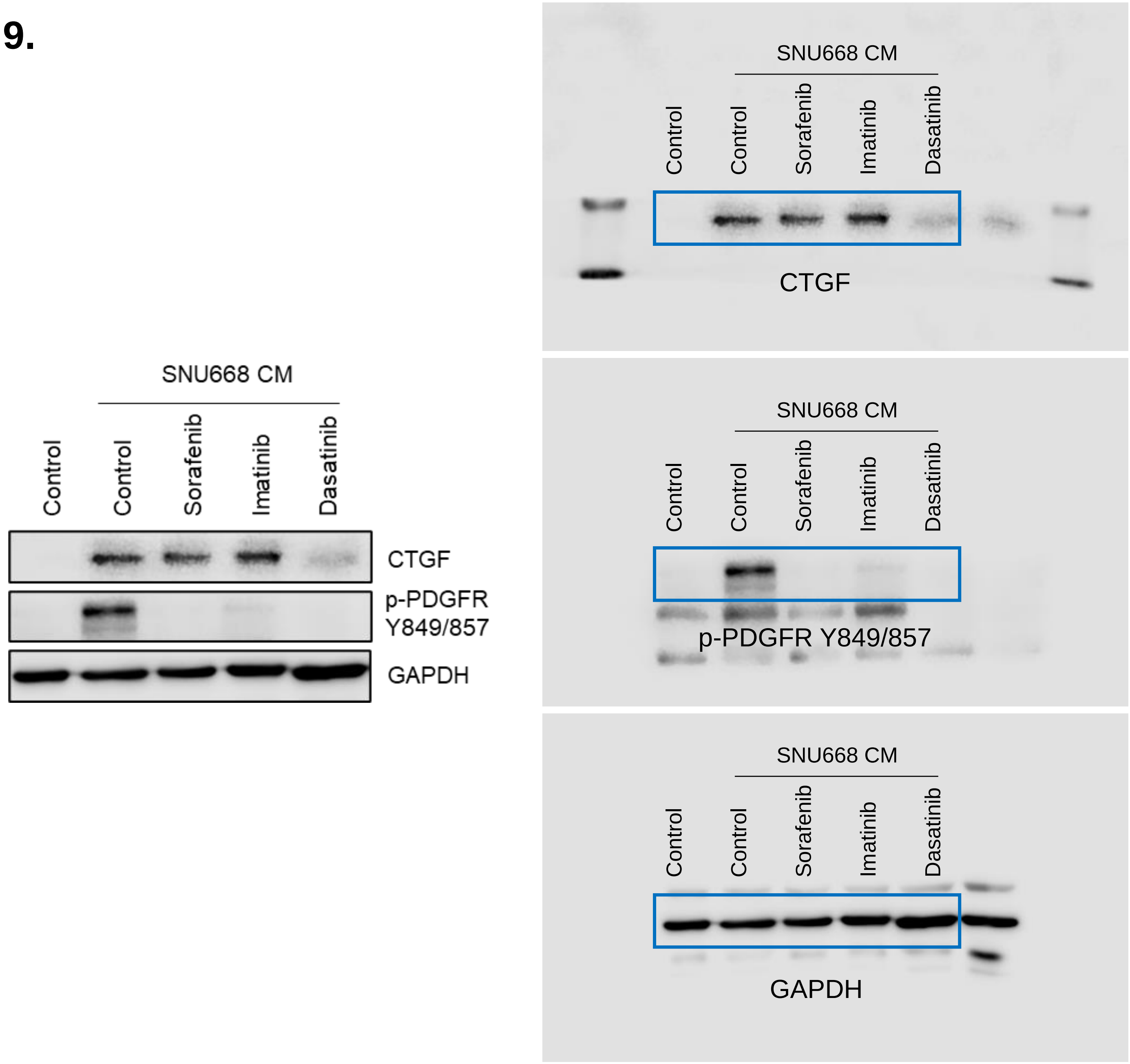

Supplementary figure 12.

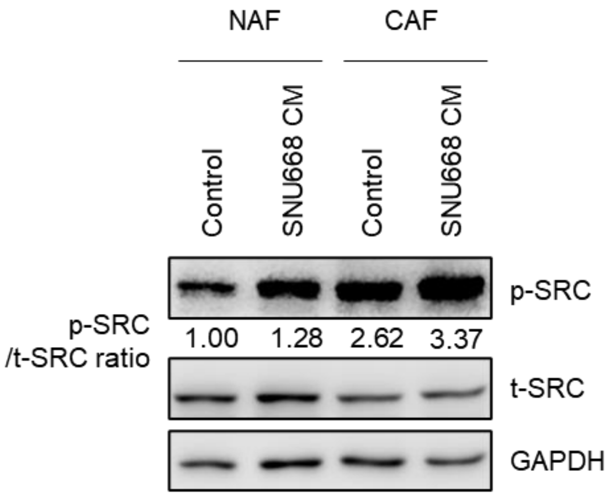

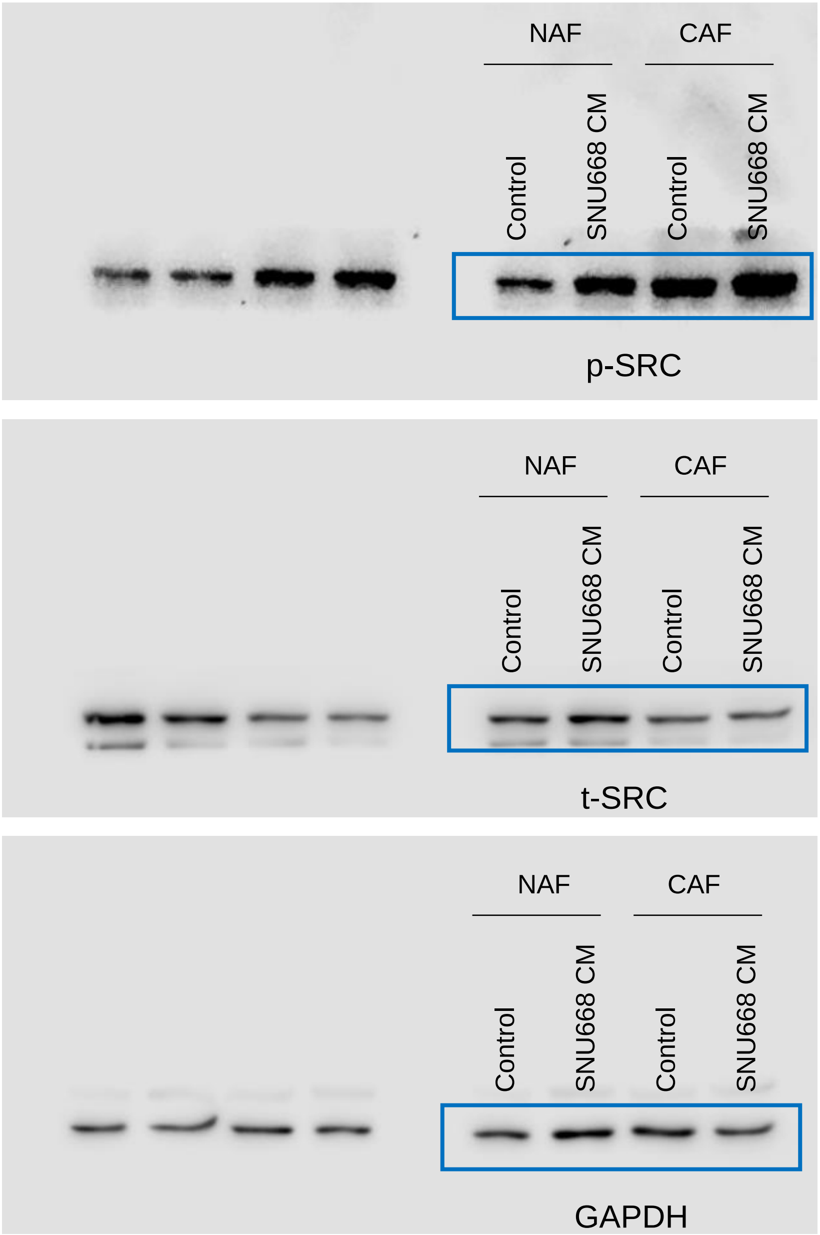

Supplementary figure 14.

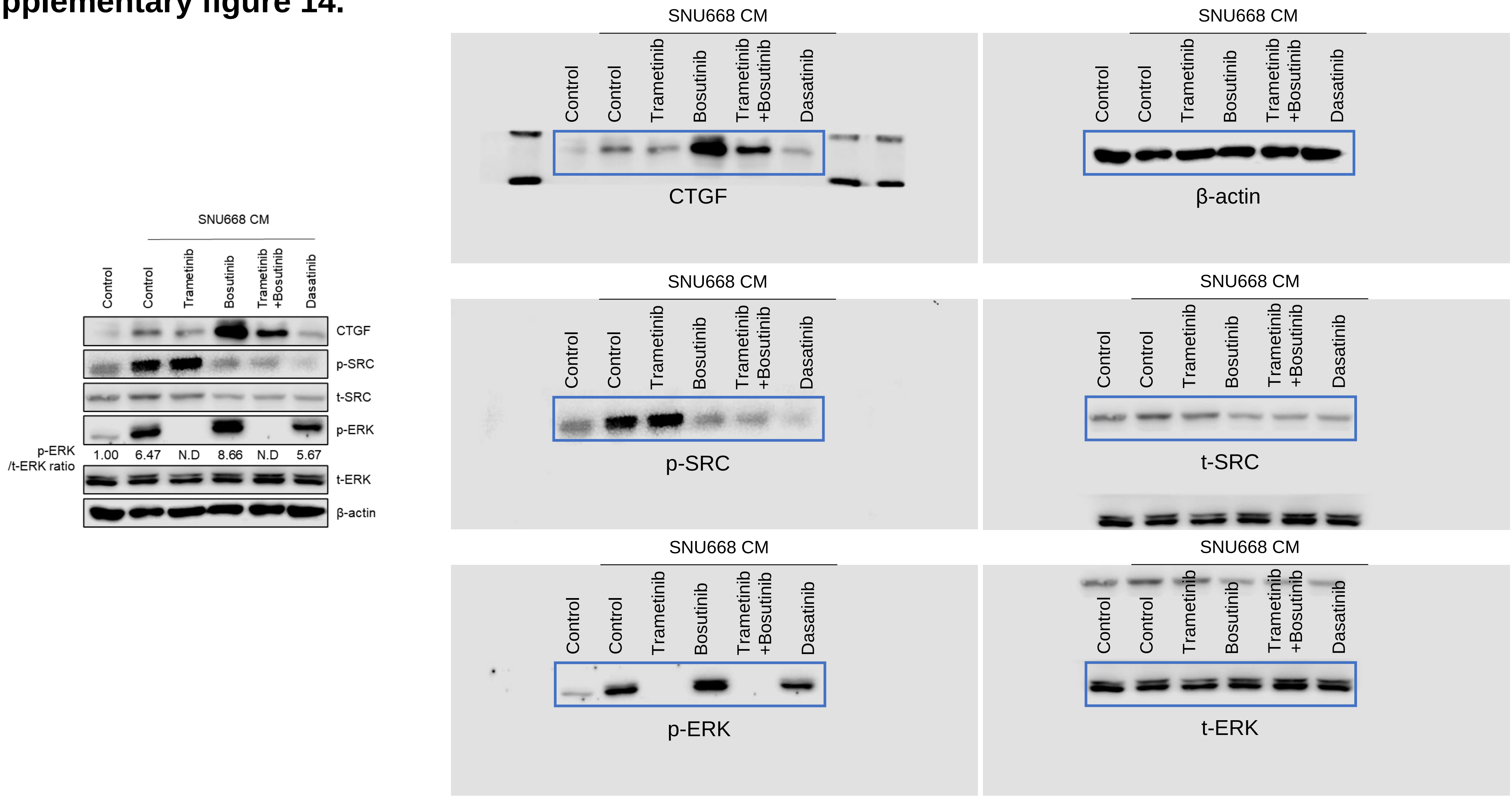

Supplement: Supplementary file 3 — Additional file 2. [file 12964_2023_1396_MOESM2_ESM.pdf]
